# The relevance of cannabinoid receptor 2 in the central nervous system: an update over the last 3 years

**DOI:** 10.3389/fnbeh.2026.1882543

**Published:** 2026-07-01

**Authors:** Camilla Moliterni, Daniela Caissutti, Enrico Mandolini, Elena Fasciolo, Tuba Rana Caglar, Antonella Capozzi, Roberta Misasi, Niccolò Candelise

**Affiliations:** 1 Department of Experimental Medicine, Sapienza University of Rome, Rome, Italy; 2 Istituto Superiore di Sanita’ ISS, Rome, Italy; 3 Department of Biology and Biotechnologies “Charles Darwin”, Sapienza University of Rome, Rome, Italy

**Keywords:** CB2R, endocannabinoid system, G protein–coupled receptor, neurodegeneration, neuroinflammation

## Abstract

The endocannabinoid system is a neuromodulatory network regulating synaptic plasticity, neuronal activity, and neuroinflammatory responses in both the central and peripheral nervous systems. The endocannabinoid system comprises endogenous ligands, termed endocannabinoids, and two principal receptors, cannabinoid receptor type 1 and type 2 (CB1R and CB2R). While CB1R is predominantly associated with the central nervous system and mediates the psychotropic effects of cannabis-derived compounds, CB2R was initially considered mainly peripheral. However, growing evidence over the last decades has highlighted a pivotal role for CB2R in central nervous system homeostasis and pathology. Importantly, the lack of psychotropic effects associated with CB2R signaling has positioned this receptor as a promising therapeutic target for several brain-related disorders, including neuroinflammatory, neurodegenerative, neuropsychiatric, and neurovascular conditions. Here, we provide a structured review of experimental studies published over the last 3 years investigating CB2R modulation in the central nervous system, with a particular focus on disease mechanisms and emerging therapeutic strategies.

## 1 Introduction

The endocannabinoid system (ECS) is a fundamental neuromodulatory network that maintains central nervous system (CNS) homeostasis by regulating synaptic plasticity, neuronal activity and neuroinflammatory processes (De Melo Reis et al., 2021). This complex signaling system comprises two primary cannabinoid receptors, cannabinoid type 1 receptor (CB1R) and cannabinoid type 2 receptor (CB2R), as well as endogenous ligands and the enzymes responsible for their synthesis and degradation (Cristino et al., 2020). CB1R is highly expressed in the CNS, where it mediates the psychotropic effects of cannabis derivatives and modulates neurotransmitter release, with lower expression in peripheral tissues. CB2R was initially characterized as primarily expressed peripherally, especially in circulating immune cells (e.g., neutrophils and monocytes) (Buckley et al., 2000; Simard et al., 2022). Increasing evidence supports CB2R expression in glial cells and in neurons within the hippocampus, nucleus accumbens, striatum, and brainstem (Van Sickle et al., 2005; Brusco et al., 2008). Neuronal CB2R was also shown to modulate dopaminergic, serotonergic and glutamatergic neurotransmission (Zhang et al., 2021; Grabon et al., 2023b; Meanti et al., 2025; Zhao et al., 2025).

**Figure 1:**
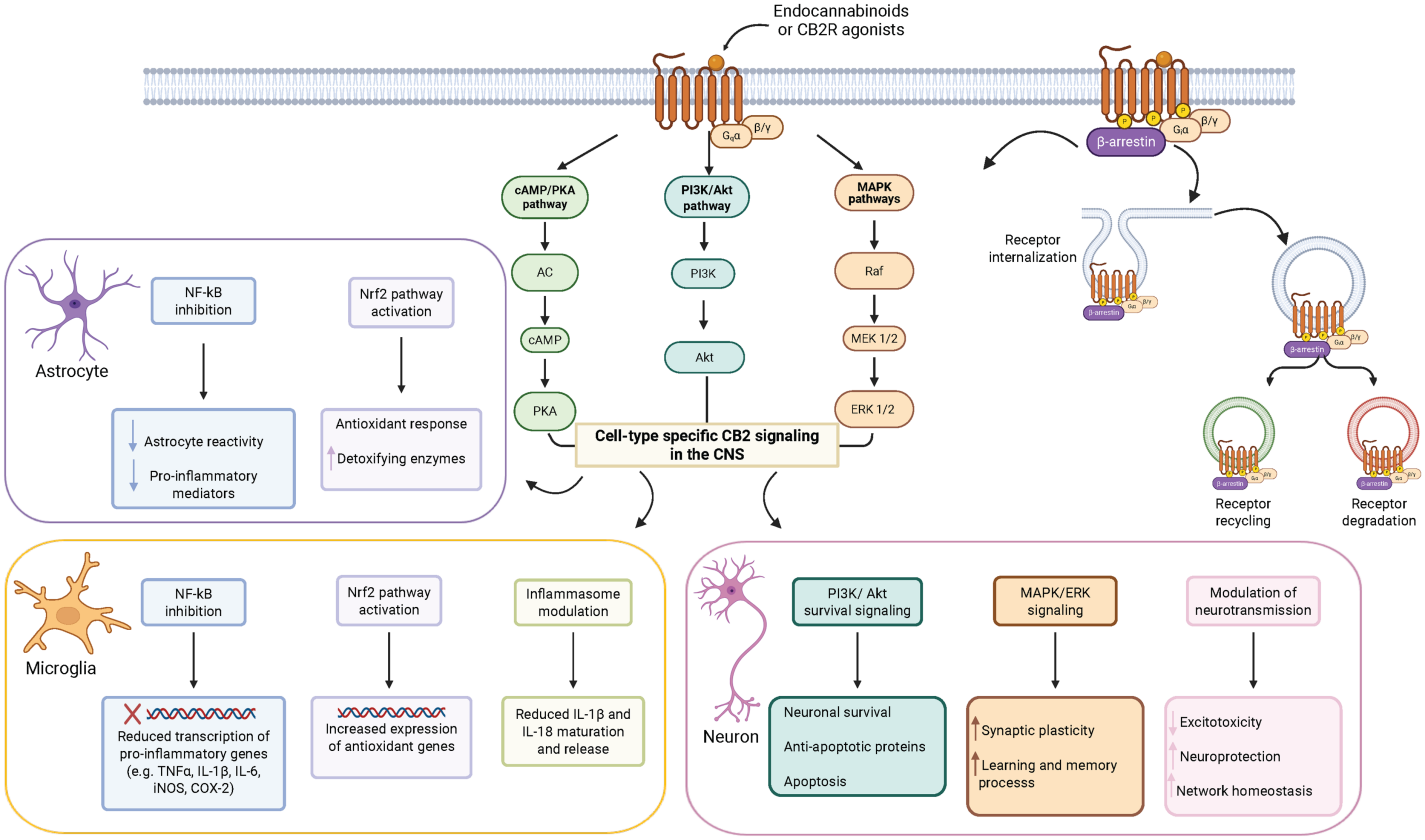
Pathways downstream CB2R activation. Schematic image showing the integrated biological activities elicited by CB2R activation, leading to NF-κB, Nrf2 and inflammasome modulation, along with arrestin-mediated recycle or degradation of the receptor. This figure has been created with BioRender.com.

CB2R is a G protein–coupled receptor, typically coupled to Gi/o proteins, and its activation inhibits adenylyl cyclase, leading to reduced intracellular cAMP levels and downstream protein kinase A activity (Demuth and Molleman, 2006). CB2R also recruits *β*-arrestins, regulating receptor desensitization, internalization and downstream inflammatory signaling (Chiurchiù et al., 2015; Soethoudt et al., 2017). Beyond this canonical pathway, CB2R signaling engages multiple intracellular signaling pathways relevant to neuroinflammation, including the PI3K/Akt and mitogen-activated protein kinase (MAPK) cascades, which regulate cell survival, cytokine production, and microglial activation. A key mechanism underlying CB2R-mediated anti-inflammatory effects is the inhibition of the NF-κB pathway, resulting in decreased expression of pro-inflammatory mediators. CB2R activation has also been associated with the induction of antioxidant responses via the Nrf2 pathway and with the modulation of inflammasome activity, contributing to the control of oxidative stress and inflammatory signaling. Collectively, these pathways place CB2R as a key regulator of neuroinflammatory responses and microglial activity. In addition, CB2R can form functional heteromers with receptors such as CB1R and CXCR4 (Callén et al., 2012; Coke et al., 2016; Scarlett et al., 2018), thereby modifying receptor coupling, downstream signaling, and cellular responses in a context-dependent manner (Figure 1).

Under physiological conditions, CB2R expression in the CNS is relatively low but becomes markedly upregulated in reactive microglia and, to a lesser extent, astrocytes and neurons following injury or inflammatory stimuli (Grabon et al., 2023a,b; Grabon et al., 2024). As the resident immune cells of the CNS, microglia undergo profound functional and transcriptional changes in response to stress, trauma, or neurodegeneration, contributing to inflammatory and oxidative processes associated with neuronal dysfunction (Gao et al., 2023). CB2R activation counteracts these responses by limiting pro-inflammatory cytokine release, reducing oxidative stress, and promoting neuroprotective microglial phenotypes (Komorowska-Müller and Schmöle, 2020).

Although microglia represent the most extensively studied CB2R-expressing CNS population, recent evidence demonstrates important functions for CB2R in astrocytes, neurons, endothelial and infiltrating immune cells. These cell populations appear to engage distinct downstream signaling programs. In microglia, CB2R primarily regulates inflammatory responses through pathways involving NF-kB, NLRP3 inflammasome activity, PI3K/Akt-Nrf2 signaling and metabolic reprogramming. In astrocytes, CB2R has been linked to autophagy, proteostasis and glial reactivity, while neuronal CB2R was shown to contribute to synaptic homeostasis, neurotransmitter release and dopaminergic, serotonergic and glutamatergic signaling. In endothelial cells, CB2R participates in the regulation of blood–brain barrier (BBB) integrity, leukocyte recruitment and vascular inflammatory responses. Together, these observations support a model in which CB2R signaling is highly cell type-dependent and shaped by the local pathological environment. Due to its immunomodulatory properties and lack of psychotropic effects, CB2R has emerged as a therapeutic target in inflammatory, autoimmune, neurodegenerative and neuropsychiatric disorders (Cabañero et al., 2021). Selective CB2R agonists thus appear promising candidates for further investigation, as they are designed to target CB2R with little or no affinity for CB1R, avoiding CB1R-mediated adverse effects and improving therapeutic applicability in inflammatory pathologies (Pertwee, 2012; Picone and Kendall, 2015; Capozzi et al., 2021).

In this review, we systematically screened the scientific literature over the last 3 years to highlight the recent advancements in the understanding of CB2R function, its involvement in CNS disorders and its potential as a therapeutic target. We focus on experimental evidence describing the mechanistic involvement of CB2R in CNS pathologies and, importantly, pain biology is not discussed, since it is often at the crossroads between the central and peripheral nervous systems and many reviews have already been produced on medical usage and anti-nociceptive effects of phytocannabinoids (Whiting et al., 2015; Finn et al., 2021; Soliman et al., 2021; Barakji et al., 2023).

## 2 Search methods, eligibility criteria, and screening

A structured literature search was performed using the PubMed database (National Institutes of Health). The search covered publications from January 1st, 2023 to April 1st, 2026, and was conducted during April 2026.

Terms were selected to capture CB2R and its gene nomenclature, including “CB2R,” “CB-2 receptor,” “CNR2,” “CNR-2,” and “cannabinoid receptor 2”. These terms were combined with pathology-specific keywords (using the Boolean operator AND/OR), corresponding to major neurological and neuropsychiatric disease categories (neuroinflammation, neurodegeneration, depression, anxiety, psychosis, schizophrenia, epilepsy, and neurovascular conditions).

The search was restricted to original research articles using PubMed filters. Reviews, editorials, conference abstracts, comments, errata, and retracted publications were excluded. Studies were further screened to retain only those providing experimental evidence on the role of CB2R within the central nervous system, with emphasis on mechanistic and preclinical investigations. Inclusion criteria comprised: (i) original experimental studies, (ii) investigation of CB2R function, and (iii) CNS-relevant models. Exclusion criteria included non-CNS studies, purely descriptive expression analyses without functional assessment, and non-English articles.

The search yielded 56 records for neuroinflammation, 38 for neurodegeneration, 17 for depression, 17 for anxiety, 11 for psychosis-related terms, 7 for schizophrenia, 7 for epilepsy, and 2 for neurovascular conditions. Study selection followed a structured screening process. After removal of duplicates and preliminary exclusions, records were screened for eligibility based on title and abstract. Full-text articles were then assessed, and studies not meeting the inclusion criteria were excluded. The final set of studies included only those providing experimental evidence on CB2R function within the central nervous system. No additional eligible studies were identified through Google Scholar searches.

## 3 Results and discussion

### 3.1 Neuroinflammation

Immune responses in the nervous system are essential for tissue homeostasis, pathogen clearance and injury repair. Excessive or unresolved inflammation drives neuronal dysfunction, synaptic impairment and tissue damage, fueling the progression of neurodegenerative and neuroinflammatory disorders (Müller et al., 2025). In this regard, CB2R is widely recognized as an important regulator of inflammatory processes in the CNS and a potential therapeutic target for neuroinflammatory and neurodegenerative diseases (Zhang et al., 2021; Grabon et al., 2023b; Meanti et al., 2025; Zhao et al., 2025). Detection of CB2R expression in the CNS remains challenging, as its expression is generally low under physiological conditions and can be dynamically regulated in response to stimuli in a time- and cell type-dependent manner (Grabon et al., 2023a,b; Grabon et al., 2024). To this end, several studies have provided important advances in the spatial and temporal characterization of tissue CB2R expression. Microglia represent the main mediators of immune surveillance and phagocytic activity in the CNS. Their activation is often categorized into classical (M1-like) or alternative (M2-like) phenotypes (Komorowska-Müller and Schmöle, 2020). M1 microglia produce pro-inflammatory cytokines and chemokines and express the NADPH oxidase, which generates reactive oxygen species (ROS) and inducible nitric oxide synthase (iNOS). M2 activation describes anti-inflammatory and healing microglial activities, releasing anti-inflammatory cytokines, growth and neurotrophic factors. Hence, CB2R signaling fine-tunes microglial activation and the balance between protective and detrimental inflammatory responses.

The literature search identified 23 CNS-focused studies investigating CB2R signaling in neuroinflammation over the last 3 years. Consistent with the central role of microglia in CNS immunity, 16 of the 23 original research articles specifically focused on this cell population.

Grabon et al. mapped the expression of CB2R across 11 brain regions by RT-qPCR in three mouse strains (C57BL/6, Balb/c, and Swiss), identifying microglia as the principal cell type contributing to CB2R expression. Under physiological conditions, CB2R expression was shown to be low and uniform across the examined regions. However, its expression was differentially regulated depending on the inflammatory stimuli. In particular, *in vitro* analyses showed that LPS-induced inflammation produced an early and transient reduction in CB2R mRNA levels in both BV2 cells and microglia isolated from C57BL/6 mice, whereas IFN-*γ* stimulation induced a rapid increase in CB2R expression in BV2 cells, supporting a stimulus-dependent regulation of CB2R expression (Grabon et al., 2024). Intriguingly, Laloli et al. developed a novel inducible microglia-specific CB2R knockout mouse model incorporating a dual reporter system, enabling simultaneous visualization of both *Cnr2* expression and gene deletion (Laloli et al., 2025). This model provides a valuable tool for investigating microglia-specific CB2R signaling. Despite the different experimental approaches, these studies converge on the low expression of CB2R under basal conditions and its dynamic regulation by inflammatory clues, supporting the concept of CB2R as a context-dependent sensor of immune activation rather than a constitutively active immunomodulatory receptor.

Multiple studies highlight the functional interplay between CB2R and microglial inflammatory pathways. Standoli et al. described a functional cross-talk between the ECS and sphingosine-1-phosphate (S1P) signaling in BV2 microglial cells. They showed that LPS induces sphingosine kinase 1 and 2 (SphK1/2), required for S1P production and downstream pro-inflammatory cytokine release. Pharmacological enhancement of endocannabinoid tone via Fatty Acid Amide Hydrolase (FAAH) inhibition or direct activation of CB2R reduced TNFα and IL-1β release and suppressed SphK1/2 induction. These findings link LPS-driven inflammation to CB2R-mediated immunomodulation (Standoli et al., 2023).

Further mechanistic insight was provided by Rodrigues et al., who investigated the role of CB2R-related signaling in the regulation of NLRP3 inflammasome activation, iNOS and microglial polarization. In BV2 cells exposed to lipopolysaccharide (LPS), CBD reduced the expression of activation markers, limiting pro-inflammatory cytokine release and promoting a shift away from a classically activated microglial phenotype. These effects were partially reversed by CB2R antagonism, indicating that CB2R-dependent signaling contributes to CBD-mediated modulation of inflammasome activity and inflammatory output, although iNOS and nitric oxide regulation appeared largely CB2R-independent (Rodrigues et al., 2024).

Consistently, studies using selective CB2R agonists further underscore its role in shaping microglial polarization. Wang et al. demonstrated that activation of CB2R with JWH133 in a cellular model of Parkinson disease inhibited pro-inflammatory M1 polarization while promoting M2-associated markers. This phenotypic switch was accompanied by activation of the PI3K/Akt pathway and enhanced Nrf2 nuclear translocation, reversed by CB2R antagonism, supporting a CB2R-dependent neuroprotective signaling cascade (Wang et al., 2023). Moreover, Chen et al. provided evidence that CB2R-mediated regulation of microglial phenotype involves Nogo-B as a downstream effector. CB2R activation with HU-308 promoted a shift from M1 toward M2 polarization, reducing TNF-*α* while increasing IL-10 production. Importantly, Nogo-B overexpression attenuated these effects, identifying it as a critical mediator of CB2R-dependent immunomodulation (Chen et al., 2025). Beyond polarization, CB2R influences microglial metabolic programming. Shan et al. showed that CB2R activation attenuates AngII-induced microglial activation and inflammatory cytokine production by suppressing aerobic glycolysis, associated with reduced neuroinflammation and improved systemic outcomes (Shan et al., 2024).

Remarkably, despite the use of different inflammatory paradigms, these studies reveal a notable mechanistic convergence. Indeed, CB2R activation consistently promoted a shift away from pro-inflammatory microglial states. PI3K/Akt-Nrf2 signaling, suppression of glycolytic metabolism, and modulation of downstream effectors such as Nogo-B all converge toward reduced cytokine production and enhanced expression of reparative markers. Together, these observations suggest that CB2R functions as an upstream regulator capable of coordinating multiple anti-inflammatory programs according to cellular context.

Consistent with these mechanistic observations, CB2R-mediated immunomodulatory effects have been described in overlapping neuroinflammatory and neurodegenerative settings. Ricardi et al. tested the immunomodulatory effect of β-caryophyllene (BCP) in the HMC3 microglial cell line exposed to Amyloid β (Aβ) peptide. In addition to attenuating Aβ cytotoxicity, BCP reduced secretion of pro-inflammatory cytokines, enhancing that of anti-inflammatory ones and regulated NF-κB activation, partially abolished by the CB2R antagonist SR144528 (Ricardi et al., 2025). Two groups explored the role of the ECS in regulating neuroinflammation in *in vivo* models of kainic acid-induced neuronal injury. Karan and colleagues showed that local injury in the dorsal hippocampus triggers a rapid neuroinflammatory response in distant, non-lesioned brain regions. At later time points, CB2R expression was upregulated in distant regions of ventral hippocampus, neocortex and cortical meninges, suggesting its involvement in delayed inflammatory responses. Pharmacological blockade of CB2R with the antagonist AM630 during injury and early post-injury significantly impaired the upregulation of the anti-inflammatory mediator Cx3CL1 in the neocortex, indicating a role for CB2R in the modulation of inflammatory responses in the CNS (Karan et al., 2024). A different pharmacological approach was employed by Arimura and co-workers, who developed a reversible inhibitor of monoacylglycerol lipase (a major hydrolase of 2-AG in the brain) and tested its efficacy in a mouse model of neuronal injury. The compound attenuated cognitive deficits and neuronal loss, modulated microglial and astrocytic activation and attenuated inflammatory signaling. This neuroprotective effect was reported to be CB2R dependent, as the effect was attenuated by AM630 (Arimura et al., 2024).

Neuroimmune regulation through the CB2R was investigated in pure inflammatory settings beyond neurodegeneration. Starr and colleagues explored the immunoregulatory role of CB2R agonism in *in vitro* systems of HIV-infected brain-resident myeloid lineage cells, such as human monocyte-derived macrophages (MDMs) and induced pluripotent stem cell-derived microglia (iMg). The CB2R selective agonist JWH-133 dose-dependently impaired HIV replication in MDMs and iMg, likely reflecting differences in baseline *Cnr2* expression, and reduced cytokine release from HIV-infected MDMs but not iMg. Transcriptomic analyses revealed that CB2R agonism primarily altered interferon and integrated stress response pathways in MDMs while altering synapse maintenance and phagocytosis in iMg. CB2R activation attenuated HIV-induced NLRP3 inflammasome activation in iMg, without decreasing NF-κB activation (Starr et al., 2025). The involvement of CB2R in systemic inflammatory conditions was explored by Matias et al. using a rat model of cecal ligation and puncture-induced sepsis, where early CB2R blockade with AM630 reduced sepsis-induced fear generalization but did not increase survival rate or alter TNF-*α* levels (Matias et al., 2023). Chen and Mackie explored neurodevelopmental inflammation using cannabinoid receptor knockout mice. They compared the role of CB2R in offspring cognitive impairment caused by perinatal cannabinoid exposure (PCE) and maternal immune activation (MIA). CB2R was shown to be involved in MIA-related neurodevelopmental deficits, as CB2R KO adult offspring did not exhibit MIA-related cognitive abnormalities. The combination of PCE and MIA produced behavioral defects only in CB2R KO adult offspring, suggesting a compensatory role for CB2R signaling under both environmental stressors (Chen and Mackie, 2025). Videtta and colleagues investigated the analgesic efficacy of an essential oil (EO) from non-psychotrophic *Cannabis sativa* in the experimental autoimmune encephalomyelitis (EAE) mouse model of multiple sclerosis. In addition to improving the overall symptomatology of EAE mice, EO induced a shift in spinal and hippocampal microglia toward an anti-inflammatory phenotype and enhanced CB2R expression. Administration of AM630 reversed the EO anti-inflammatory response in LPS-stimulated BV-2 microglial cells and abolished its anti-allodynic effect in EAE mice, linking these effects to CB2R pathways (Videtta et al., 2026).

Since long-term alcohol intake is known to induce neuroinflammation (Adams et al., 2023) in both humans and animal models, the role of CB2R in alcohol-induced neuroinflammation has been investigated using conditional knockout (cKO) approaches. Roberts and colleagues used microglia-specific cKO mice and observed that the selective KO of CB2R in microglia under basal conditions and during alcohol administration enhanced the expression of pro-inflammatory cytokines TNF-*α*, IL-6, IL-1α, and IL-1β in the hippocampus of mice (Roberts et al., 2023). Consistently, Kibret and coworkers employed cKO mice with selective deletion of CB2R in dopamine neurons and in microglia. Cell type-specific CB2R deletion significantly increased pro-inflammatory cytokine levels in the prefrontal cortex, striatum, and hippocampus. Treatment with the non-selective cannabinoid receptor mixed agonist WIN 55,212–2 significantly reduced alcohol preference compared to the vehicle controls, further linking CB2R signaling to alcohol-induced neuroinflammation (Kibret et al., 2023). As microglia-driven neuroinflammation is a key pathogenic mechanism of depression and cognitive disorders as well (see the dedicated paragraph below) (Xia et al., 2025), Wang et al. explored the CB2R-mediated anti-inflammatory effect of the antidepressant agent esketamine (ESK), resulting in decreased pro-inflammatory cytokine and nitrite levels, along with downregulated iNOS and NF-κB signaling in BV-2 microglial cells exposed to LPS. AM630 reversed the modulatory effect of ESK on microglial activation, suggesting that the CB2R-mediated immunoregulatory effects may also be involved in the antidepressant effect of ESK (Wang et al., 2024).

Beyond mood-related neuroinflammation, CB2R signaling has also been implicated in inflammation-associated cognitive dysfunction. Wu et al. linked decreased oleamide levels to more severe post-operative cognitive dysfunction and increased inflammatory markers in socially isolated mice. By upregulating hippocampal expression of FoxQ1, a transcription factor involved in inflammatory regulation and cellular stress responses, oleamide-mediated CB2R activation restored cognitive function and ameliorated neuroinflammation (Wu et al., 2025). Similarly, Chen and colleagues investigated the involvement of BCP in mitigating neuroinflammation in a mouse model of perioperative neurocognitive disorders, comprising cognitive impairments caused by surgery and anesthesia associated with microglia-mediated neuroinflammation (Chen et al., 2023). These mice treated with BCP showed increased CB2R expression 24 h after surgery in the hippocampus and a reduction of neuroinflammation as evidenced by decreased IL-1β and IL-6 levels. Co-administration of AM630 and BCP attenuated these anti-inflammatory effects in response to microglia activation, supporting the immunomodulatory role of CB2R in attenuating neuroinflammation and microglial activation. Although microglia represent the primary CB2R-expressing immune cells in the CNS, recent studies have demonstrated that CB2R expression is also selectively upregulated in brain endothelial cells in response to inflammatory stimuli, suggesting a direct involvement of this receptor in the vascular response to injury (Ramirez et al., 2012). Using a mouse model of traumatic brain injury, Bullock and colleagues explored *Cnr2* expression in brain endothelial cells, showing that CB2R was selectively upregulated under inflammatory conditions. Pharmacological activation of this receptor with the selective agonist PM289 on the hCMEC/D3 endothelial cell line preserved blood–brain barrier integrity, reduced TNF-*α*–induced ICAM-1 expression and inhibited NF-κB signaling, further supporting a protective role for CB2R in neuroinflammation-associated vascular dysfunction (Bullock et al., 2023).

The vast majority of recent studies support a predominantly anti-inflammatory role for CB2R signaling across diverse experimental settings. Despite differences in species, disease models, and pharmacological approaches, these studies consistently report reduced cytokine production, attenuation of microglial activation, and preservation of tissue homeostasis following CB2R activation. However, emerging evidence suggests that this framework may not be universally applicable.

Indeed, in contrast to the abovementioned literature, Moe et al. highlighted a detrimental pro-inflammatory role of microglial CB2R signaling in graft-versus-host disease (GVHD). Using a murine model of GVHD, the authors showed that CB2R expression is upregulated in activated microglia and contributes to CNS inflammation. Notably, genetic deletion of host CB2R reduced microglial activation, donor T-cell infiltration, and neuronal injury, indicating a key role for CB2R in GVHD-associated neuroinflammation. Consistently, pharmacological blockade of CB2R with the brain-penetrant inverse agonist/antagonist SMM-189 attenuated CNS inflammation without affecting systemic disease, further supporting the involvement of microglial CB2R signaling in immune cell recruitment and neuroinflammatory processes during GVHD (Moe et al., 2024).

Overall, recent evidence identifies microglial CB2R as a central regulator of neuroimmune homeostasis. Across pharmacological, genetic, and disease-specific models, CB2R signals repeatedly converge on three major biological processes: modulation of inflammatory cytokine production, regulation of microglial activation state and control of cellular metabolic programs. Nevertheless, the observation that CB2R blockade can also exert beneficial effects in selected pathological settings, such as GVHD, indicates that receptor function is strongly influenced by disease context and cellular environment. Thus, rather than acting as a uniformly anti-inflammatory receptor, CB2R appears to behave as a dynamic modulator of immune responses, whose effects depend on the underlying pathological state.

### 3.2 Neurodegenerative disorders

Recent evidence points to the ECS as a relevant player in neurodegenerative diseases, with CB2R as a promising pharmacological target, largely due to its anti-inflammatory effects and lack of psychotropic activity (Chen et al., 2017; Lu and Mackie, 2021; Reyes-Resina et al., 2025). Our search yielded 37 papers over the last 3 years for neurodegeneration. Targeted search for Alzheimer disease (AD) and Parkinson disease (PD) resulted in 17 and 14 entries, respectively, while one entry each was found for Huntington disease (HD) and Frontotemporal dementia (FTD). After screening for studies focused on the central activity of CB2R, we selected 8 papers addressing AD, 7 PD, 2 FTD, and 4 other scientific works related to neurodegenerative conditions, presented together in this section.

#### 3.2.1 Alzheimer disease

AD is the most common form of dementia (Sobue et al., 2024; Lui and Tsao, 2026), characterized by progressive cognitive decline, memory loss and behavioral changes. Its pathophysiological hallmarks include cholinergic deficiency, Aβ pathology, tau protein hyperphosphorylation and neuroinflammation (Schwab et al., 2022). Among the many pathogenic mechanisms involved in AD, the ECS has emerged as a potential therapeutic target due to its ability to modulate major neurotransmission pathways related to AD (Basavarajappa et al., 2017). Recent studies consistently support CB2R upregulation in AD-associated neuroinflammatory environments, particularly in relation to Aβ pathology, glial activation and cognitive decline. Pharmacological activation of CB2R generally exerts neuroprotective effects across both genetic and pharmacological AD models, although sex-dependent and cell-specific differences have also emerged.

Medina-Vera and co-workers sought to assess CB2R expression levels in an App^NL-G-F^ knock-in mouse model of AD. CB2R gene expression was found to be upregulated in 6- and 12-month-old App^NL-G-F^ mice, while remaining low at 2 months of age, in parallel with Aβ pathology, suggesting a correlation between CB2R expression and disease severity. Immunostaining experiments confirmed the presence of CB2R in different brain regions including astrocytes surrounding Aβ deposits. Primary cultures of neurons and astrocytes derived from wild-type (WT) mice exposed to Aβ42 showed increased CB2R levels, while cultures from App^NL-G-F^ mice showed even higher CB2R expression upon treatment, further supporting the association between Aβ pathology and CB2R upregulation (Medina-Vera et al., 2023).

Sobue and co-workers employed the same mouse model and reported increased CB2R mRNA expression in microglia from 8-month-old App^NL-G-F/NL-G-F^ mice. Building on previous findings showing elevated CB2R expression in the hippocampus, frontal and temporal cortex of postmortem human AD brains (Benito et al., 2003; Cavanna and Trimble, 2006; Galán-Ganga et al., 2020) and the established role of CB2R in neuroinflammation, the group investigated whether the selective CB2R agonist JWH133 could ameliorate cognitive impairments. Object recognition tests showed improved memory performance following repeated JWH133 administration. Furthermore, chronic JWH133 administration led to a reduction of dystrophic neurites in the cerebral cortex, a well-established feature of AD pathology (Sadleir et al., 2016), indicating that CB2R stimulation improve cognitive performance and reduce neuritic damage in the App^NL-G-F/NL-G-F^ mice (Sobue et al., 2024).

Tisi and co-workers employed 12-month-old Tg2576 mice overexpressing the amyloid precursor protein (APP)—a stage preceding hippocampal Aβ plaque formation—to investigate ECS alterations in the retina. CB2R expression was found to be significantly upregulated compared to WT mice. Immunofluorescence on retinal cryosections revealed increased CB2R signal across all retinal layers, along with enhanced IBA-1 signal, indicating microglial activity, while GFAP staining showed no significant differences between groups. The widespread distribution of CB2R upregulation across retinal layers suggests the involvement of multiple cell types, in agreement with previous reports in AD patient brains (Tisi et al., 2025).

Pacheco-Sanchez and co-workers exploited the 3 × Tg-AD mouse model, to assess *Cnr2* gene expression in primary hippocampal astrocyte cultures from female mice. Lower *Cnr2* levels were detected in AD compared to WT astrocytes, with female WT astrocytes showing higher *Cnr2* expression, highlighting sex-dependent differences in cannabinoid signaling that should be considered when investigating AD pathogenesis (Pacheco-Sánchez et al., 2023).

The search for multi-target pharmacological strategies was pursued by Hu and co-workers, who investigated the effects of *Guilingji* (GLJ), a traditional Chinese herbal medicine formulation comprising over 20 components, on the APPswe/PS1ΔE9 transgenic AD mouse model. GLJ administration reduced Aβ deposition in the hippocampus and attenuated neuronal apoptosis as assessed by immunostaining, Nissl and TUNEL analyses. GLJ improved long-term memory performance, suggesting a differential impact on specific memory processes. *In vitro* experiments on HT22 and BV2 cell lines further supported its potential as a multi-target pharmacological approach in AD (Hu et al., 2025).

Zhu and co-workers demonstrated that extracellular vesicle (EV)-mediated delivery of the CB2R agonist AM1241 delayed neurodegeneration and improved neuronal function recovery in APP/PS1 mice. EVs-AM1241-treated mice showed learning performance comparable to WT animals in the Morris water maze and fear conditioning test, along with electrophysiological recordings indicating higher activity in hippocampal CA1 units. Histological analyses revealed reduced Aβ plaque burden in the cortex and hippocampus, decreased BACE1 expression, reduced apoptosis, and increased expression of neuronal markers NeuN and Tuj1. Mechanistically, calcium-Erk signaling pathways were found to be regulated following EVs-AM1241 treatment, supporting EV-mediated CB2R agonist delivery as a therapeutic strategy (Zhu Y. et al., 2023).

Beyond genetic models, pharmacological approaches represent an additional avenue for AD modeling and ECS modulation. Toledano and Akirav employed the intracerebroventricular streptozotocin (ICV-STZ) rat model of sporadic AD to evaluate the effects of chronic CBD administration. CBD prevented STZ-induced impairments in the Object Location and Novel object recognition tests. CB2R mRNA expression was upregulated in the hippocampus of ICV-STZ rats, an effect prevented by CBD treatment in both the CA1 and dentate gyrus regions. Co-administration of the CB2R antagonist AM630 abolished the behavioral benefits of CBD, indicating partial CB2R involvement in CBD-mediated protection (Toledano and Akirav, 2025). Finally, Spatz and co-workers relied on a pharmacological model of AD based on oligomerized A*β*_25-35_ peptide injection to evaluate a series of merged human butyrylcholinesterase inhibitor/CB2R ligands. One compound dose-dependently attenuated Aβ_25-35_ -induced learning deficits in both the Y-maze spontaneous alternation task and the passive avoidance response test, supporting a dual-target approach in AD (Spatz et al., 2023).

Together, AD studies point toward a consistent framework in which CB2R expression increases alongside disease progression and neuroinflammatory burden. Despite the heterogeneity of experimental approaches, interventions targeting CB2R ultimately reduce glial activation, attenuate neuroinflammation and improve cognitive outcomes. Hence, the therapeutic potential of CB2R in AD primarily derives from its ability to modulate the neuroimmune environment rather than directly targeting amyloid pathology. Still, whether CB2R-mediated neuroprotection results predominantly from microglial, astrocytic or neuronal signaling remains incompletely resolved and warrants further investigation.

#### 3.2.2 Parkinson disease

PD is the most common neurodegenerative movement disorder (Li et al., 2025), characterized by the loss of dopaminergic neurons of the substantia nigra pars compacta, causing bradykinesia, rigidity, tremor and postural instability (Poewe et al., 2017). Given the potential of CB2R modulation to influence neuroinflammation and neurodegeneration, research has focused on identifying pathways that could offer therapeutic benefit without the psychotropic effects associated with CB1R activation.

Receptor-level interactions were explored by Reyes-Resina and colleagues, who investigated the CB2R-NMDAR heterodimer in the context of PD. Using HEK-293 T cells expressing either or both receptors and exposed to *α*-synuclein (α-syn) fibrils, the authors showed that whereas CB2R agonist JWH133 normally decreased intracellular cAMP and induced β-arrestin II recruitment, pre-exposure to α-syn fibrils attenuated both effects. Cross-antagonism events between CB2R and NMDAR were further confirmed in primary microglial cultures, where α-syn fibrils reduced the heteromer abundance and modulated downstream signaling through both the cAMP and MAPK pathways. Notably, CB2R agonism upregulated M2 microglial markers while downregulating M1 markers, underscoring the neuroprotective role of the ECS. These findings suggest that CB2R-NMDAR complexes may play a relevant role in α-synucleinopathies (Reyes-Resina et al., 2025).

The neuroprotective potential of CB2R activation was further supported by Liu and colleagues, who employed two rat models of PD—the 6-OHDA model and primary ventral mesencephalon neurons treated with MPP+. In both models, administration of JWH133 restored key markers of neurodegeneration, tyrosine hydroxylase-positive neuron count and mitochondrial membrane potential while reducing ROS accumulation, effects reversed by co-treatment with the CB2R antagonist AM630. The group also reported that JWH133 normalized the Bcl-2/Bax ratio and reduced cleaved caspase-3 expression, indicating an anti-apoptotic action. Additionally, JWH133 partially reversed MPP + -induced dysregulation of iron transport proteins DMT1 and FPN1, suggesting that CB2R activation may reduce iron accumulation and neurodegeneration (Liu et al., 2024). A different perspective was offered by Dos Santos Pereira and colleagues, who assessed the role of CB2R in L-DOPA-induced dyskinesia (LID) in hemiparkinsonian mice. Although selective CB2R agonists failed to rescue LID manifestation, the semisynthetic cannabidiol derivative 4′-F-CBD combined with Chlorpromazine produced a partial reduction in striatal astrocyte activation and attenuated the upregulation of corticostriatal glutamatergic synaptic markers vGluT1 and PSD95 in 6-OHDA lesioned mice treated with L-DOPA, suggesting a normalization of overactive glutamatergic transmission (Dos Santos Pereira et al., 2024). The role of astrocytic CB2R was specifically addressed by Zhu and colleagues (Zhu H. et al., 2023) in a subacute 1-methyl-4-phenyl-1,2,3,6-tetrahydropyridine (MPTP) model of PD. Selective knockdown of CB2R in astrocytes exacerbated MPTP-induced TH neuron loss in the substantia nigra pars compacta, while pre-administration of JWH133 improved motor impairments and dopamine levels and reduced astrocyte activation. The authors showed that these effects depended on the autophagic response in astrocytes, consistent with the central role of autophagy and proteostasis in neurodegeneration models (Tanaka and Matsuda, 2014; Hipp et al., 2019; Candelise et al., 2023; Montalesi et al., 2025).

Transcriptomic sequencing revealed that FOXG1 protein was upregulated in LPS, ATP- and MPP + treated primary astrocytes, reversed by CB2R activation. RT-qPCR analyses further confirmed that JWH133 promoted MAP1LC3B mRNA levels, leading the authors to propose that CB2R activation inhibits the interaction between FOXG1 and MAP1LC3B, hence promoting MAP1LC3B transcription. FOXG1 knockdown was produced in a PD-like mouse model treated with JWH133, and both behavioral assessment and immunohistopathological analyses were performed. FOXG1 knockdown abolished the neuroprotective effects of JWH133, linking CB2R signaling to autophagic regulation and protein quality control in PD (Zhu H. et al., 2023).

The modulation of *α*-syn pathology by CB2R was investigated by two additional groups. Joers and colleagues used a rat model of synucleinopathy based on AAV2/5-mediated human α-syn overexpression and found that systemic administration of SMM-189, a CB2R inverse agonist, reduced phosphorylated α-syn at Ser129. Feng and colleagues, on the other hand, employed CB2R KO mice injected with fibrillar α-syn in the nucleus accumbens, demonstrating that CB2R deficiency prolonged microglial activation, exacerbated synaptic pruning and enhanced cholinergic synapse loss (Feng et al., 2023). Mechanistically, CB2R deficiency potentiated CREB phosphorylation and c-fos expression downstream of α-syn stimulation, a pathway shown to drive complement-mediated microglial engulfment of synaptic elements (Joers et al., 2024).

Finally, Targa and colleagues examined CB2R involvement in sleep and memory alterations in a rotenone model of PD. Intrastriatal infusion of the AM630 reversed rotenone-induced changes in sleep macrostructure and interhemispheric desynchronization. At the same time, the partial agonist GW405833 improved object recognition memory, suggesting that sleep and cognitive alterations in PD involve partially independent CB2R-dependent mechanisms (Targa et al., 2025).

Remarkably, in contrast to AD studies, recent PD works highlight the role of CB2R as a regulator of both neuroinflammation and dopaminergic neuronal survival. On the one hand, this dual activity may suggest that CB2R occupies a strategic position in PD, where inflammatory and neurodegenerative processes are tightly interconnected; on the other hand, this discrepancy may suggest that neuronal CB2R signaling deserves further investigation in AD, as it could represent an overlooked factor contributing to the complexity of this disease.

#### 3.2.3 Frontotemporal dementia and other neurodegenerative conditions

FTD is a heterogeneous group of early-onset, progressive neurodegenerative disorders characterized by degeneration of the frontal and temporal lobes, causing progressive cognitive, behavioral and language impairment (Boeve et al., 2022). Two major molecular classes account for approximately 95% of clinical FTD cases: FTD-TAU, related to misfolded Tau protein in neurons, and FTD-TDP-43, characterized by intraneuronal cytoplasmic aggregates of TDP-43 (Cairns et al., 2007; Riku et al., 2022; Silva-Llanes et al., 2025). The role of CB2R modulation in FTD has been investigated across both molecular subtypes, yielding mechanistically distinct findings. Silva-Llanes and colleagues focused on FTD-TAU, examining how the CB2R antagonist PGN36 affects cognitive decline in a Tau-dependent mouse model. Tau^P301L^ overexpression was induced in the hippocampus via viral injection, followed by administration of PGN36 or vehicle. Building on prior findings from the same group, which demonstrated that CB2R deficiency ameliorates cognitive impairment induced by Tau^P301L^ overexpression (Galán-Ganga et al., 2021), behavioral testing confirmed that TAU^P301L^ overexpression induces cognitive impairment, while PGN36 administration restored the discrimination index to control levels, indicating that CB2R modulates Tau^P301L^-induced cognitive decline. Tau^P301L^ overexpression further induced *Cnr2* expression, an effect reversed by PGN36 treatment. Importantly, PGN36 did not affect *Cnr1* expression, confirming the compound selectivity for *Cnr2*. Tau^P301L^ overexpression broadly downregulated gene expression, with most transcripts normalized following PGN36 administration. Pathway enrichment analysis identified synapse organization and signaling among the most significantly affected cellular functions. Immunofluorescence assessment of brain-derived neurotrophic factor (BDNF) levels in the CA3 hippocampal region confirmed these findings, while in the dentate gyrus, PGN36 failed to rescue BDNF levels. DAPI staining of the DG revealed a loss of the granule layer in the TAU^P301L^ overexpression group, partially reduced by PGN36 treatment (Silva-Llanes et al., 2025).

In a CaMKIIα-TDP43 mouse model of FTD-TDP-43, Gonzalo-Consuegra and colleagues evaluated the CB2R agonists HU-308 and RO-6866945. Both compounds rescued the impaired discrimination and preference indexes observed in untreated transgenic mice during the Novel Object Recognition test. In the medial prefrontal cortex, CB2R agonists restored corticospinal motor neuron immunoreactivity and reduced astrogliosis and microgliosis, assessed by S-100β and Iba1 staining, respectively. Similar effects were observed in the hippocampal CA1 region, while in the dentate gyrus, neuronal signal recovery was accompanied by reduced glial activation. Importantly, phosphorylated TDP-43, a hallmark of protein aggregation and proteostatic stress (Tan et al., 2017; Candelise et al., 2021; Lu et al., 2025), was elevated in CaMKIIα-TDP43 mice and reduced following RO-6866945 treatment, supporting a role for CB2R activation in mitigating proteostatic pathology in FTD (Gonzalo-Consuegra et al., 2024).

Notably, the divergent responses observed between FTD-TDP43 and FTD-Tau models illustrate a broader theme emerging throughout the CB2R literature: receptor modulation cannot be universally classified as beneficial or detrimental. Instead, therapeutic outcomes appear strongly dependent on the underlying molecular pathology, emphasizing the importance of disease-specific approaches when targeting CB2R. Research on CB2R involvement in neurodegeneration has also been extended to models of HD. Paredes-Ruiz and colleagues assessed the role of a selective monoacylglycerol lipase inhibitor, JZL184, in 3-nitropropionic acid (3-NP)-induced mitochondrial dysfunction, a well-established model recapitulating several hallmarks of HD in cellular and *in vivo* contexts. 3-NP induced a 55% reduction in mitochondrial activity, which was completely rescued by JZL184 administration. In synaptosome preparations, this protective effect was reversed by the CB2R inverse agonist JTE907, suggesting a CB2R -dependent mechanism of action (Paredes-Ruiz et al., 2023). Employing the same 3-NP model, Kordinová and colleagues synthesized and biologically evaluated a series of 2,6,9-trisubstituted purine derivatives (compounds displaying selective CB2R agonist activity) in differentiated SH-SY5Y neuron-like cells. The tested compounds exhibited neuroprotective effects by modulating both caspase-dependent and caspase-independent apoptotic pathways, with one derivative emerging as the most effective, combining CB2R agonist activity with pronounced cytoprotective effects (Kordinová et al., 2026).

Parallel efforts have been directed toward the development of novel synthetic ligands with enhanced selectivity for CB2R over CB1. Gioé-Gallo and colleagues evaluated the neuroprotective potential of a series of newly developed compounds in primary mouse cells and in retinoic acid- and GLP-1-differentiated SH-SY5Y human neuroblastoma cells transiently transfected with MAPT P301L and APP V717I expression plasmids. Across both models, the compounds demonstrated neuroprotective effects that were consistently reversed by the CB2R antagonist SR144528, confirming CB2R-mediated action (Gioé-Gallo et al., 2025).

The protective role of CB2R was further explored in a rotenone-induced cytotoxicity model by Rathod and Agrawal, who investigated the effects of the natural compound BCP in SH-SY5Y cells. Assessment of the GSK3β/Nrf2/HO-1 signaling axis revealed that rotenone increased GSK3β activity while decreasing Nrf2 and HO-1 activity, effects partially reversed by BCP treatment (Rathod and Agrawal, 2025). Overall, these studies emphasize two recurring mechanistic themes: the tight association between CB2R upregulation and pathological protein accumulation, and the capacity of CB2R signaling to modulate neuroimmune responses across diverse disease contexts. While most studies support a neuroprotective role for receptor activation, emerging disease-specific differences indicate a strong dependence on the underlying molecular pathology, affected cell populations and stage of disease progression.

### 3.3 Neuropsychiatric disorders

The ECS is widely recognized to play a central role in neuropsychiatric disorders, particularly in affective disorders such as depression and anxiety (Stampanoni Bassi et al., 2018; Chadwick et al., 2020; Gallego-Landin et al., 2021). Depressive disorders were reported to affect 3.8% of the global population, with higher prevalence in women (Otte et al., 2016). Due to the therapeutic potential of ECS modulation in psychiatric disorders, research has focused on identifying pathways that could elude the psychotropic effects mediated by the CB1R. Compared to neurodegenerative disorders, evidence linking CB2R to neuropsychiatric phenotypes remains more heterogeneous and mechanistically fragmented, mostly relying on behavioral paradigms and pharmacological modulation.

The systematic search for research papers that investigated CB2R in depression and anxiety yielded 17 entries for each topic over the last 3 years. Independent searches for “psychot*,” “epilepsy” and “schizophrenia” were conducted to cover the most common neuropsychiatric disorders associated with CB2, yielding 11, 7 and 7 entries, respectively.

After screening for experimental works focused on CNS-relevant CB2R activity, we selected 4 papers for the “depression” group, 6 for the “anxiety” group, and 3 from the other groups, presented together in this section.

Depression and anxiety can be evaluated in WT murine models by established behavioral tests. Hen-Shoval and co-workers (Hen-Shoval et al., 2023) assessed depressive-like behavior using the Forced Swim Test (FST) in female and male Wistar-Kyoto rats, a strain displaying depressive- and anxiety-like phenotypes (Malkesman et al., 2006; Seedat et al., 2009). Animals were acutely treated with Cannabidiolic Acid Methyl-Ester, a semi-synthetic analogue of the CBD precursor Cannabidiolic Acid. This compound was shown to exert antidepressant-like effects in all genders in terms of decreased immobility and increased swimming during the FST. Notably, the antidepressive effects were abolished by pre-treatment with AM630 only in female rats. The CB2R antagonist further prevented the down-regulation of FAAH, the key catabolic enzyme for endocannabinoids, supporting a sex-dependent involvement of CB2R signaling.

The pharmacological modulation of the ECS was employed by Kruk-Slomka and co-workers (Kruk-Slomka et al., 2025) to assess anxiety behavior in male Swiss mice using the Elevated Plus Maze test. Here, a battery of CB2R agonists and antagonists was injected intraperitoneally (i.p.) and behavior was assessed following acute administration. Acute CB2R modulation induced anxiogenic-like effects, in terms of reduced entries and time spent in the open arms of the maze. Although apparently conflicting with part of the previous literature, the authors discussed the possibility of an opposite effect between acute and chronic administration of CB2R ligands, still confirming the central role of CB2R in emotional behavior. Together, these findings highlight a context-dependent role of CB2R in affective behavior, influenced by sex, pharmacological profile, and timing of receptor modulation.

A similar pharmacological approach was put forth by Johnson and colleagues (Johnson et al., 2023) using the zebrafish *Danio rerio* as an experimental model. The authors sought to investigate, using the Open Field Test, the potential anxiolytic effects of terpenes with a similar mode of action to cannabinoids and addressed the involvement of CB receptors using selective ligands. Behavioral analyses showed that terpenes BCP and Terpinolene reduced zebrafish anxiety-like behavior, reversed by AM630, suggesting a CB2R-mediated effect. BCP was also shown to affect hedonic behavior in female Swiss mice (Dos Santos Barbosa et al., 2023), mechanistically linked to CB2R activity through pre-administration of AM630. Researchers observed reduced motivational salience in food-fasted mice treated with BCP and the CB2R agonist JWH-133 during the runway task and conditioned place preference test, prevented by pre-treatment with AM630. These results extend CB2R involvement to motivation and reward processing.

Besides Terpenes, the search for compounds that can act on affective disorders by modulating the ECS was expanded by Wang and co-workers (Wang et al., 2025) to the traditional functional Chinese medicine plant *Schisandra chinensis*. In this work, researchers applied the established Chronic Unpredictable Mild Stress paradigm to induce depressive behavior in male C57BL/6 mice. Animals received oral administration of control solution or *Schisandra chinensis* extract and received i.p. administration of the CB2R agonist WIN55212-2 and the CB2R antagonist AM630. Whereas the plant extract was able to reduce depressive-like symptoms induced by the unpredictable stress paradigm, the protective effect was abolished by CB2R blockade, which further enhanced microglial phagocytic activity, supporting the central role of CB2R in brain-resident immune cells.

A different pharmacological approach was taken by Yang and co-workers (Yang et al., 2024). Researchers designed various CBD derivatives with potential dual activity on CB2R and the serotonergic receptor 5-HT1A and selected the one showing the most favorable brain exposure. Low acute doses of the compound produced antidepressive effects as assessed by FST and Tail-Suspension Test (TST), while higher concentrations did not affect the behavior. Higher doses were, however, required to exert anxiolytic effects on stress-induced hypothermia, suggesting a differential concentration effect of the compound acting on CB2R and 5-HT1A. Insights on the interaction between CB2R and the neurotransmission system were further reported by Canseco-Alba and colleagues (Canseco-Alba et al., 2024). Researchers employed a conditional double KO of CB2R and the Dopamine Transporter DAT on a C57BL/6 background. Mice were treated with methamphetamine to model schizophrenia-like behavior, observing a reduced percentage of pre-pulse inhibition and reduced social preference, supporting the relevance of midbrain-expressed CB2R in models of psychosis.

Neuroanatomical evidence has been obtained by Uzuneser and co-workers (Uzuneser et al., 2023) by targeting the brain-specific transporter of endocannabinoids, Fatty Acid Binding protein 5 (FABP-5), through direct injection in the prelimbic region of male Sprague Dawley rats, challenged with antagonists of the ECS. The FABP-5 inhibitor exerted dose-dependent anxiolytic effects, reversed by AM630. Electrophysiological recordings revealed that FABP-5 inhibition caused the attenuation of firing rate from the medial prefrontal cortex to the basolateral amygdala, elevated the firing rate of ventral hippocampal neurons, and altered the local field potential of both brain regions. These effects were reversed by AM630, indicating that the neural circuitry governing anxiety behavior might be modulated by CB2R signaling.

Whereas the reported works employ highly heterogeneous experimental paradigms, they collectively support the shared mechanism by which CB2R signaling regulates behavior through neuroimmune pathways. Since depressive and anxiety-like phenotypes are frequent comorbidities across neurological disorders, many pre-clinical studies assessed them as symptoms of different experimental setups involving CB2R in the CNS.

In a model of chronic epilepsy induced by pilocarpine (Cai et al., 2024), CB2R agonist AM1241 was shown to alleviate epileptic seizures and associated depression, as demonstrated by FST, TST and sucrose preference test. Mice displaying depressive-like behavior after seizure induction showed increased hippocampal expression of CB2R. Co-immunofluorescence staining of the receptor with the microglial marker Iba1 showed that CB2R expression was predominantly localized in microglia, suggesting a strong link between neuroinflammation and ECS activity through CB2R. Accordingly, the agonist AM1241 reduced seizure frequency and depressive behavior, along with reduced neuronal loss in the hippocampal CA3 region and reduced neuroinflammation. Notably, these studies reinforce the link between CB2R activity and neuroimmune modulation, particularly through microglial responses, as a shared mechanism across distinct pathological contexts.

Two recent studies investigated CB2R signaling in alcohol-related neuropsychiatric phenotypes (Gasparyan et al., 2023; Navarrete et al., 2025), frequently related to anxiety behavior (American Psychiatric Association, 2013). In their first work, they evaluated the anxiolytic action of CBD after spontaneous alcohol withdrawal, observing an increased expression of the gene *Cnr2* in the *nucleus accumbens*, potentiated by CBD administration. A similar increase of *Cnr2* was reported, in their second work, in a model of Fetal Alcohol Spectrum Disorder. Mice displayed reduced time in the lighted box and increased immobility in the TST, substantiating the relevance of CB2R in the regulation of emotional behaviors. Early life adversity was further related to depressive behavior and reported to be rescued by increased CB2R activity through lentiviral prelimbic injection in female rats after maternal separation (Andersen, 2024), substantiating the importance of CB2R signaling in higher-order brain regions.

Expression of CB2R was shown to be modulated during unhealthy aging as well by Jantsch and co-workers (Jantsch et al., 2025). Here, aged male Wistar rats fed with an obesogenic diet displayed anxiety-like behavior when tested using the Elevated Plus Maze, a phenotype partially rescued by CBD administration. Notably, cafeteria diet caused the reduction of CB2R transcripts in the prefrontal cortex, further reduced by CBD administration, thus supporting the involvement of ECS dysregulation in anxiety-related phenotypes during unhealthy aging.

Overall, CB2R is gaining increased attention as a potential target for neuropsychiatric disorders. Recent evidence positions CB2R as a context-dependent modulator of affective and psychotic phenotypes, acting through neuroimmune and broader neuromodulatory mechanisms. Pharmacological approaches have revealed the central role of this receptor in the modulation of emotional behavior. Across models, CB2R effects converge on three main axes: (i) modulation of endocannabinoid tone (e.g., FAAH), (ii) regulation of neuroimmune responses via microglia, and (iii) interaction with classical neurotransmitter systems, particularly dopaminergic and serotonergic pathways. As the neurobiology of psychiatric disorders is multi-factorial, future work is needed to better understand the involvement of the ECS and CB2R. While neuroimmune mechanisms currently represent the strongest and most consistent evidence base, increasing data suggest that CB2R may influence higher-order behavioral functions through coordinated effects on both immune and neurotransmitter networks. Indeed, CB2R may influence behavioral phenotypes not only through neuroimmune regulation but also through direct and indirect modulation of monoaminergic signaling networks. Furthermore, gender differences appear to be key in both psychiatric disorders and ECS regulation, yet few recent works have addressed the differences in their experimental setup. Furthermore, multiple behavioral tests seem to be required to evaluate nuances in behavioral output, both in murine and alternative models. Future studies should integrate behavioral analyses with molecular and biochemical characterization of ECS components. Overall, the development of selective CB2R-targeting compounds may improve the management of major neuropsychiatric disorders and related comorbidities.

### 3.4 Neurovascular disorders

Neurovascular disorders comprise pathological conditions affecting the cerebral vasculature, including ischemic stroke and cerebrovascular inflammatory damage. The ECS plays a pivotal role in protecting both neurons and vessels, with both cannabinoid receptors expressed in neurons and endothelial cells, regulating the permeability of the BBB and neuroinflammation.

Our systematic literature search over the last 3 years yielded 9 results for “ischemia” and 2 for “neurovascular,” which were reduced to 3 studies after removal of duplicates and exclusion of non-CNS-related works.

Numerous studies have confirmed the significant neuroprotective effect of the plant-derived CB2R agonist BCP in cerebral ischemia (Flores-Soto et al., 2021; Mallmann et al., 2022; Zhang et al., 2022; Zhao et al., 2023). Notably, the role of BCP in protecting against white matter damage was recently evaluated. White matter, largely composed of myelinated axons, is particularly vulnerable to ischemic damage (Wang et al., 2016) due to limited blood flow and reduced collateral circulation. As a result, axonal glial disruption and demyelination may occur, representing a key factor contributing to cognitive dysfunction following cerebral ischemia (Rosenzweig and Carmichael, 2015). Therefore, in the work of Xin et al., the authors used a mouse model of middle cerebral artery occlusion and oligodendrocyte OGD/R (oxygen and glucose deprivation/reperfusion) models to explore the role of BCP in white matter damage and repair after ischemic stroke (Xin et al., 2024). Overall, they found that in mice, after ischemic stroke, BCP treatment improved motor and cognitive functions through CB2R activation and preserved Myelin-associated glycoprotein and myelin basic protein expression levels, consistent with its protective effects on white matter integrity following ischemic stroke. These findings support a role for BCP in preserving white matter integrity after cerebral ischemias. They further showed in cultured oligodendrocytes that BCP is able to increase their viability and reduce LDH release, by attenuating OGD/R-induced cellular damage and NLRP3-mediated pyroptosis (Xin et al., 2024).

A model of cerebral ischemia–reperfusion injury (CIRI) was deployed by Li and co-workers (Li et al., 2024) to assess the effect of the CB2R selective agonist AM1241 on alleviating this kind of injury. Researchers observed reduced neuroinflammation and oxidative stress both in microglial BV2 cells and in a mouse model of CIRI after treatment with AM1241, substantiating its role as a neuroprotective agent. Notably, they linked the biological effect to the interaction of AM1241 with the complex of the Toll-like receptor 4 (TLR4) and myeloid differentiation factor 2 (MD2), as demonstrated by co-immunoprecipitation and MD2 overexpression in cells. Surface Plasmon Resonance experiments reported a dissociation constant of 8.27 μM for AM1241 and MD2, substantially higher than the dissociation constant of AM1242 with the CB2R (Yao et al., 2006). Hence, the contribution of CB2R signaling relative to MD2/TLR4 interaction should be further clarified before attributing these effects to non-canonical AM1241 activity. Nevertheless, these findings warrant further investigation into potential off-target or CB2R-independent effects of AM1241.

He and colleagues investigated whether physical exercise could enhance the microglial protection in a CB2R- and P2Y12-dependent manner (He et al., 2025). P2Y12, a purinergic receptor highly expressed in homeostatic microglia, is dramatically reduced after microglial activation (Yu et al., 2019). Preservation of P2Y12 expression may therefore represent a strategy to maintain protective microglia–neuron interactions during ischemic injury. Their data demonstrated that PE increased microglial P2Y12 expression, enhancing microglial dynamics and promoting microglial contacts with neurons as assessed by *in vivo* two-photon imaging. The effect of physical exercise has been correlated to the activity of CB2R, since it increased CB2R expression in microglia. Furthermore, CB2R activation was involved in increasing Nrf2 phosphorylation and MafK transcription factor level, ultimately upregulating P2Y12 expression. Thus, PE-induced CB2R signaling may support neuronal protection by preserving microglia–neuron somatic junctions (He et al., 2025).

Collectively, these findings support CB2R activation as a potential therapeutic strategy against ischemic and neurovascular damage.

## 4 Conclusion and future perspectives

**Figure 2:**
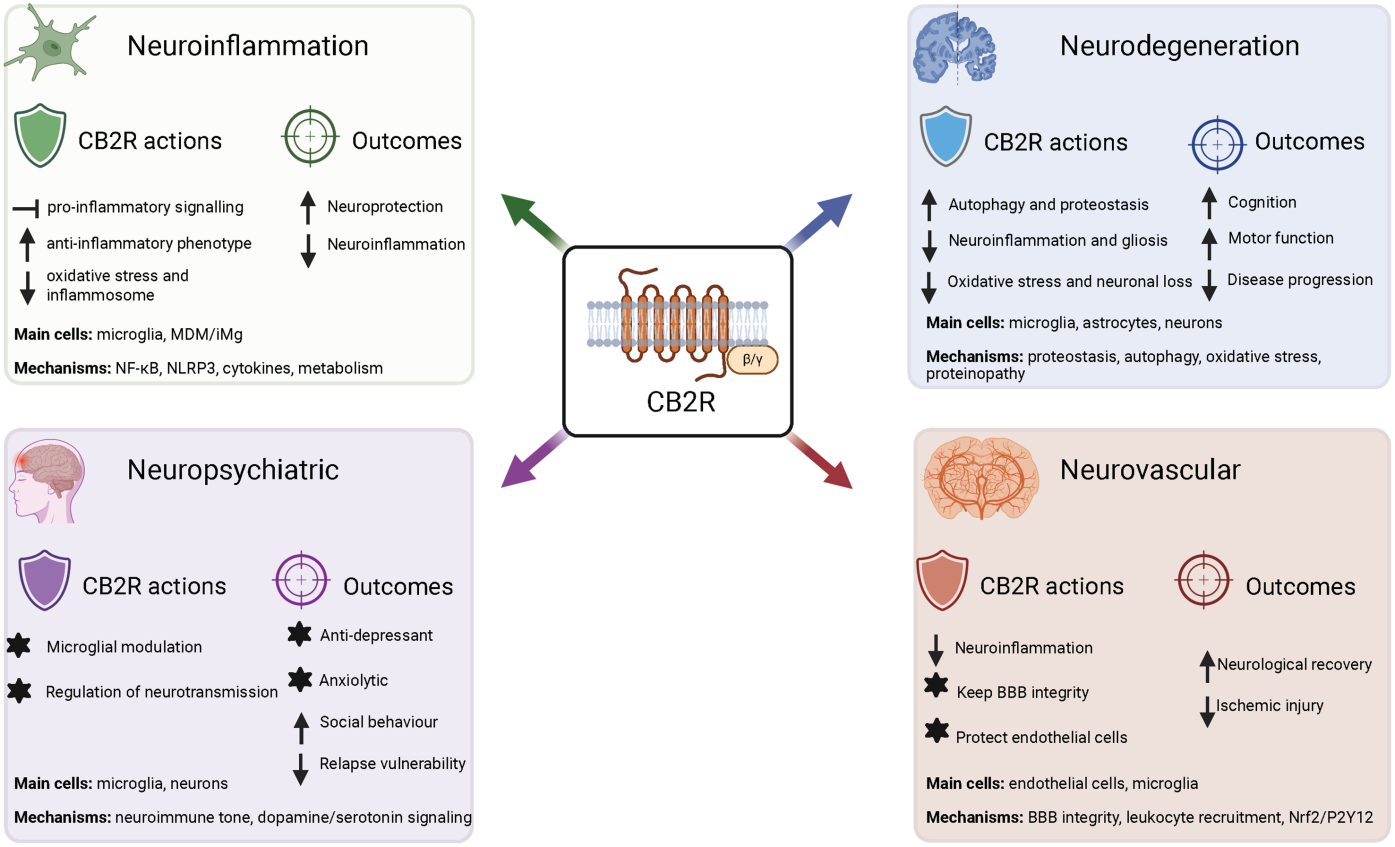
CB2R involvement in main neurological disorders. Recapitulatory scheme showing CB2R action and outcomes in major CNS disorders. This figure has been created with BioRender.com.

Over the last 20 years, there has been a surge in scientific papers that investigated the role of CB2R in the CNS, growing from 30 entries in 2006 to more than 60 publications per year over the last 5 years, according to a broader “neuro* “search along with the working prompt. Advancements have been made in the understanding of the importance of the ECS in most brain-related disorders, spanning from neuroinflammation and neurodegeneration to neuropsychiatric conditions, as summarized in Figure 2. An important theme emerging from recent studies is the progressive expansion of CB2R biology beyond its established role in neuroimmune regulation. While CB2R-mediated modulation of microglial activation and inflammatory responses remains consistent across many neurological models, recent works have highlighted additional functions involving astrocytic proteostasis, neuronal homeostasis, neurotransmission and neurovascular regulation. Different pathological settings appear to engage distinct CB2R-dependent programs, a concept exemplified by the divergent responses in FTD-TDP43 and FTD-Tau models, where CB2R agonism and antagonism, respectively, produced beneficial outcomes. Similar observations have been reported in GVHD-associated neuroinflammation, where CB2R blockade reduced microglial activation, immune cell infiltration and neuronal injury. Collectively, these findings indicate that the biological consequences of CB2R modulation are closely linked to the cellular and molecular landscape of each pathological condition, including the predominant cell populations involved, the inflammatory milieu and the underlying proteinopathy. In this view, CB2R emerges as a context-dependent regulator integrating neuroimmune, metabolic and synaptic responses across CNS disorders.

Of note, most evidence derives from heterogeneous experimental approaches, ranging from immortalized cell lines to conditional knockout models and cell type-specific genetic manipulations. While these approaches support a role for CB2R in CNS homeostasis, conclusions based solely on pharmacological modulation should be interpreted cautiously until confirmed through complementary experimental strategies. Pharmacological modulation of CB2R has shown promising neuroprotective and cognitive effects across CNS disorders. The main modulators of CB2R employed in recent works are listed in Table 1. Molecular actions of CB2R extend far beyond canonical Gi/o signaling, involving dynamic regulation of inflammasome activity, cellular metabolism, receptor heteromerization, and glia–neuron communication in a disease- and cell-specific manner. Once addressed, these variables, together with a better understanding of species-dependent differences in CB2R biology and pharmacological tools, may contribute to closing the gap between experimental findings and future clinical translation. Rather than acting as a simple anti-inflammatory switch, CB2R emerges as a highly plastic signaling platform whose molecular outputs are shaped by cellular identity, receptor interactome, inflammatory state, and ligand-dependent signaling bias.

**Table 1:** List of modulators of CB2R activity.

Compound	CB2R action	Model	Main effects	Mechanisms	Reference(s)
JWH133	Selective agonist	AD, PD, neuroinflammation	Improved cognition, reduced microglial activation, neuroprotection	PI3K/Akt, Nrf2 activation, M2 polarization	Sobue et al. (2024), Wang et al. (2023), Liu et al. (2024), and Zhu H. et al. (2023)
HU-308	Selective agonist	FTD-TDP43	Reduced gliosis and pTDP-43 pathology	Anti-inflammatory signaling	Gonzalo-Consuegra et al. (2024)
RO-6866945	Selective agonist	FTD-TDP43	Improved NOR performance, reduced pTDP-43	Modulation of glial activation	Gonzalo-Consuegra et al. (2024)
AM1241	Selective agonist	Epilepsy, ischemia, AD-related models	Reduced seizures, neuroinflammation, oxidative stress	CB2R-dependent immunomodulation	Cai et al. (2024), Li et al. (2024), and Zhu Y. et al. (2023)
GW405833	Partial agonist	PD	Improved recognition memory	Modulation of dopaminergic signaling	Targa et al. (2025)
WIN55,212–2	CB1R/CB2R agonist	Alcohol-related neuroinflammation, depression models	Reduced alcohol preference, antidepressant-like effects	ECS modulation	Kibret et al. (2023) and Wang et al. (2025)
CBD	Indirect/partial CB2R-related effects	AD, neuroinflammation	Cognitive rescue, reduced inflammasome activation	NF-κB inhibition, inflammasome modulation	Rodrigues et al. (2024) and Toledano and Akirav (2025)
CBDA-ME	CBR2-related activity	Depression	Antidepressant-like effects	FAAH modulation	Hen-Shoval et al. (2023)
β-Caryophyllene (BCP)	Selective agonist	AD, ischemia, anxiety, neurodegeneration	Anti-inflammatory, anxiolytic, neuroprotective	NF-κB, Nrf2/HO-1 modulation	Ricardi et al. (2025), Johnson et al. (2023), Dos Santos Barbosa et al. (2023), Xin et al. (2024), and Rathod and Agrawal (2025)
Oleamide	CB2R-related signaling	Post-operative cognitive dysfunction	Cognitive rescue, reduced neuroinflammation	FoxQ1 signaling	Wu et al. (2025)
Esketamine	CB2R-related indirect modulation	Neuroinflammation/depression	Reduced cytokine production	NF-κB/iNOS inhibition	Wang et al. (2024)
JZL184	Indirect ECS enhancer	Huntington-related models	Restored mitochondrial activity	Increased 2-AG signaling	Paredes-Ruiz et al. (2023)
FAAH inhibitors	Indirect ECS enhancer	Neuroinflammation	Reduced cytokine release	Increased endocannabinoid tone	Standoli et al. (2023)
PM289	Selective agonist	TBI/endothelial inflammation	Preserved BBB integrity	NF-κB inhibition	Bullock et al. (2023)
PGN36	Antagonist	FTD-TAU	Improved cognition	Synaptic pathway modulation	Silva-Llanes et al. (2025)
SMM-189	CB2R inverse agonist	GVHD, synucleinopathy	Reduced neuroinflammation and α-syn pathology	Regulation of microglial activation	Moe et al. (2024) and Joers et al. (2024)
SR144528	Antagonist	Multiple models	Reversed CB2R-mediated effects	Pharmacological validation	Ricardi et al. (2025) and Gioé-Gallo et al. (2025)
AM630	Antagonist	Multiple models	Blocked protective effects of agonists	CB2R validation tool	Karan et al. (2024), Arimura et al. (2024), Wang et al. (2024), Chen et al. (2023), Johnson et al. (2023), and Toledano and Akirav (2025)
Guilingji (GLJ)	Multi-component herbal formulation	AD (APPswe/PS1ΔE9 mice)	Reduced Aβ deposition, improved long-term memory, reduced apoptosis	Multi-target neuroprotection, ECS-related modulation	Hu et al. (2025)
*Schisandra chinensis* extract	Multi-component herbal formulation	Depression/CUMS model	Reduced depressive-like behavior	CB2R-dependent microglial modulation	Wang et al. (2025)

Therefore, the complexity of CB2R biology underscores the need for careful experimental design. A recurrent limitation across recent studies is the lack of mechanistic depth in many CB2R-related works, particularly in neuropsychiatric studies, where behavioral findings are often insufficiently supported by mechanistic ECS analyses. Future work should therefore prioritize the clarification of receptor- and context-specific CB2R mechanisms, addressing variables such as sex differences, disease stage, and model selection, with the ultimate goal of translating these findings into viable therapeutic strategies for human CNS-related pathologies.

## Glossary

ECS: Endocannabiniod System
CBR1/2: cannabinoid receptor 1/2
CNS: Central NErvous System
MAPK: mitogen activated protein
ROS: reactive oxygen species
iNOS: inducible nitric oxide synthase
S1P: sphingosine-1-phosphate
SphK1/2: sphingosine kinase 1/2
FAAH: Fatty Acid Amide Hydrolase
LPS: lipopolysaccharide
BCP: β-caryophyllene
Aβ: Amyloid-β
MDM: monocyte-derived macrophages
iMg: induced pluripotent stem cell-derived microglia
PCE: perinatal cannabinoid exposure
MIA: maternal immune activation
EO: essential oil
EAE: experimental autoimmune encephalomyelitis
cKO: conditional knockout
ESK: esketamine
GVHD: graft-versus-host disease
AD: Alzheimer Disease
PD: Parkinson Disease
FTD: Frontotemporal dementia
HD: Huntington Disease
WGT: wild-type
APP: Amyloid precursor protein
GLJ: Guilingji
EV: Extracellular vesicles
ICV-STZ: intracerebroventricular streptozotocin
*α*-syn: α-synuclein
LID: L-DOPA-induced dyskinesia
MPTP: 1-methyl-4-phenyl-1,2,3,6-tetrahydropyridine
BDNF: brain-derived neurotrophic factor
3-NP: 3-nitropropionic acid
FST: forced swim test
i.p.: intraperitoneal
TST: tail suspension test
FABP-5: Fatty Acid Binding protein 5
BBB: blood–brain barrier
OGD/R: oxygen and glucose deprivation/reperfusion
CIRI: cerebral ischemia–reperfusion injury
TLR4: Toll-like receptor 4
MD2: myeloid differentiation factor 2

## References

[ref1] Adams C. Perry N. Conigrave J. Hurzeler T. Stevens J. Yacou Dunbar K. P. . (2023). Central markers of neuroinflammation in alcohol use disorder: a meta-analysis of neuroimaging, cerebral spinal fluid, and postmortem studies. Alcohol. Clin. Exp. Res. 47, 197–208. doi: 10.1111/acer.14992, 36852781

[ref2] American Psychiatric Association (2013). Diagnostic and Statistical Manual of Mental Disorders. 5th Edn. Arlington, VA: American Psychiatric Association.

[ref3] Andersen S. L. (2024). Increasing CB2 receptor activity after early life stress prevents depressive behavior in female rats. Biomolecules 14:464. doi: 10.3390/biom14040464, 38672480 PMC11047932

[ref4] Arimura N. Maeda C. Aoyama K. Yamaguchi N. Sugiura A. Takahashi Y. . (2024). Compound 4f, a novel brain-penetrant reversible monoacylglycerol inhibitor, ameliorates neuroinflammation, neuronal cell loss, and cognitive impairment in mice with kainic acid-induced neurodegeneration. PLoS One 19:e0312090. doi: 10.1371/journal.pone.0312090, 39570850 PMC11581214

[ref5] Barakji J. Korang S. K. Feinberg J. Maagaard M. Mathiesen O. Gluud C. . (2023). Cannabinoids versus placebo for pain: a systematic review with meta-analysis and Trial sequential analysis. PLoS One 18:e0267420. doi: 10.1371/journal.pone.0267420, 36716312 PMC9886264

[ref6] Basavarajappa B. S. Shivakumar M. Joshi V. Subbanna S. (2017). Endocannabinoid system in neurodegenerative disorders. J. Neurochem. 142, 624–648. doi: 10.1111/jnc.14098, 28608560 PMC5669051

[ref7] Benito C. Núñez E. Tolón R. M. Carrier E. J. Rábano A. Hillard C. J. . (2003). Cannabinoid CB_2_ receptors and fatty acid amide hydrolase are selectively overexpressed in Neuritic plaque-associated glia in Alzheimer’s disease brains. J. Neurosci. 23, 11136–11141. doi: 10.1523/JNEUROSCI.23-35-11136.2003, 14657172 PMC6741043

[ref8] Boeve B. F. Boxer A. L. Kumfor F. Pijnenburg Y. Rohrer J. D. (2022). Advances and controversies in frontotemporal dementia: diagnosis, biomarkers, and therapeutic considerations. Lancet Neurol. 21, 258–272. doi: 10.1016/S1474-4422(21)00341-0, 35182511

[ref9] Brusco A. Tagliaferro P. Saez T. Onaivi E. S. (2008). Postsynaptic localization of CB2 cannabinoid receptors in the rat hippocampus. Synapse 62, 944–949. doi: 10.1002/syn.20569, 18798269

[ref10] Buckley N. E. McCoy K. L. Mezey E. Bonner T. Zimmer A. Felder C. C. . (2000). Immunomodulation by cannabinoids is absent in mice deficient for the cannabinoid CB(2) receptor. Eur. J. Pharmacol. 396, 141–149. doi: 10.1016/s0014-2999(00)00211-9 10822068

[ref11] Bullock T. A. Galpayage Dona K. N. U. Hale J. F. Morales P. Jagerovic N. Andrews A. M. . (2023). Activation of CB2R by synthetic CB2R agonist, PM289, improves brain endothelial barrier properties, decreases inflammatory response and enhances endothelial repair. NeuroImmune Pharmacol. Therapeutics 2, 387–400. doi: 10.1515/nipt-2023-0016, 38116176 PMC10726734

[ref12] Cabañero D. Martín-García E. Maldonado R. (2021). The CB2 cannabinoid receptor as a therapeutic target in the central nervous system. Expert Opin. Ther. Targets 25, 659–676. doi: 10.1080/14728222.2021.1971196 34424117

[ref13] Cai Y. Tong F. Li K. Wang Q. Ding J. Wang X. (2024). Cannabinoid receptor 2 agonist AM1241 alleviates epileptic seizures and epilepsy-associated depression via inhibiting neuroinflammation in a pilocarpine-induced chronic epilepsy mouse model. Mol. Cell. Neurosci. 130:103958. doi: 10.1016/j.mcn.2024.103958 39151841

[ref14] Cairns N. J. Bigio E. H. Mackenzie I. R. A. Neumann M. Lee V. M.-Y. Hatanpaa K. J. . (2007). Neuropathologic diagnostic and nosologic criteria for frontotemporal lobar degeneration: consensus of the consortium for frontotemporal lobar degeneration. Acta Neuropathol. 114, 5–22. doi: 10.1007/s00401-007-0237-2, 17579875 PMC2827877

[ref15] Callén L. Moreno E. Barroso-Chinea P. Moreno-Delgado D. Cortés A. Mallol J. . (2012). Cannabinoid receptors CB1 and CB2 form functional heteromers in brain. J. Biol. Chem. 287, 20851–20865. doi: 10.1074/jbc.M111.335273, 22532560 PMC3375509

[ref16] Candelise N. Caissutti D. Zenuni H. Nesci V. Scaricamazza S. Salvatori I. . (2023). Different chronic stress paradigms converge on endogenous TDP43 cleavage and aggregation. Mol. Neurobiol. 60, 6346–6361. doi: 10.1007/s12035-023-03455-z, 37450246 PMC10533643

[ref17] Candelise N. Scaricamazza S. Salvatori I. Ferri A. Valle C. Manganelli V. . (2021). Protein aggregation landscape in neurodegenerative diseases: clinical relevance and future applications. Int. J. Mol. Sci. 22:6016. doi: 10.3390/ijms22116016, 34199513 PMC8199687

[ref18] Canseco-Alba A. Tabata K. Momoki Y. Tabassum T. Horiuchi Y. Arinami T. . (2024). Cannabinoid CB2 receptors and hypersensitivity to methamphetamine: vulnerability to schizophrenia. Prog. Neuro-Psychopharmacol. Biol. Psychiatry 130:110924. doi: 10.1016/j.pnpbp.2023.110924, 38135096 PMC10872318

[ref19] Capozzi A. Caissutti D. Mattei V. Gado F. Martellucci S. Longo A. . (2021). Anti-inflammatory activity of a CB2 selective cannabinoid receptor agonist: signaling and cytokines release in blood mononuclear cells. Molecules 27:64. doi: 10.3390/molecules27010064, 35011295 PMC8746368

[ref20] Cavanna A. E. Trimble M. R. (2006). The precuneus: a review of its functional anatomy and behavioural correlates. Brain 129, 564–583. doi: 10.1093/brain/awl004, 16399806

[ref21] Chadwick V. L. Rohleder C. Koethe D. Leweke F. M. (2020). Cannabinoids and the endocannabinoid system in anxiety, depression, and dysregulation of emotion in humans. Curr. Opin. Psychiatry 33, 20–42. doi: 10.1097/YCO.0000000000000562, 31714262

[ref22] Chen F. Bai N. Yue F. Hao Y. Wang H. He Y. . (2023). Effects of oral β-caryophyllene (BCP) treatment on perioperative neurocognitive disorders: attenuation of neuroinflammation associated with microglial activation and reinforcement of autophagy activity in aged mice. Brain Res. 1815:148425. doi: 10.1016/j.brainres.2023.148425, 37244603

[ref23] Chen D. Gao M. Gao F. Su Q. Wu J. (2017). Brain cannabinoid receptor 2: expression, function and modulation. Acta Pharmacol. Sin. 38, 312–316. doi: 10.1038/aps.2016.149, 28065934 PMC5342669

[ref24] Chen S. Li Z. Yang L. Xu Z. Liu A. He Q. . (2025). Cannabinoid Receptor-2 alleviates Sepsis-induced Neuroinflammation by modulating microglia M1/M2 subset polarization through inhibiting Nogo-B expression. Mol. Neurobiol. 62, 9258–9270. doi: 10.1007/s12035-025-04836-2, 40102346

[ref25] Chen H.-T. Mackie K. (2025). CB1 and CB2 receptors differentially modulate the cognitive impact of maternal immune activation and perinatal cannabinoid exposure. Behav. Brain Res. 485:115543. doi: 10.1016/j.bbr.2025.115543, 40113177 PMC11986805

[ref26] Chiurchiù V. Battistini L. Maccarrone M. (2015). Endocannabinoid signalling in innate and adaptive immunity. Immunology 144, 352–364. doi: 10.1111/imm.12441, 25585882 PMC4557672

[ref27] Coke C. J. Scarlett K. A. Chetram M. A. Jones K. J. Sandifer B. J. Davis A. S. . (2016). Simultaneous activation of induced Heterodimerization between CXCR4 chemokine receptor and cannabinoid receptor 2 (CB2) reveals a mechanism for regulation of tumor progression. J. Biol. Chem. 291, 9991–10005. doi: 10.1074/jbc.M115.712661, 26841863 PMC4859002

[ref28] Cristino L. Bisogno T. Di Marzo V. (2020). Cannabinoids and the expanded endocannabinoid system in neurological disorders. Nat. Rev. Neurol. 16, 9–29. doi: 10.1038/s41582-019-0284-z, 31831863

[ref29] De Melo Reis R. A. Isaac A. R. Freitas H. R. De Almeida M. M. Schuck P. F. Ferreira G. C. . (2021). Quality of life and a surveillant endocannabinoid system. Front. Neurosci. 15:747229. doi: 10.3389/fnins.2021.747229, 34776851 PMC8581450

[ref30] Demuth D. G. Molleman A. (2006). Cannabinoid signalling. Life Sci. 78, 549–563. doi: 10.1016/j.lfs.2005.05.055, 16109430

[ref31] Dos Santos Barbosa L. A. Dutra R. C. Moreira E. L. G. de Carvalho C. R. (2023). β-Caryophyllene, a cannabinoid receptor 2 agonist, decreases the motivational salience and conditioning place preference for palatable food in female mice. Addict. Biol. 28:e13249. doi: 10.1111/adb.13249, 36577722

[ref32] Dos Santos Pereira M. Dias de Abreu G. H. Vanderlei L. C. A. Raisman-Vozari R. Guimarães F. S. Lu H.-C. . (2024). 4′-fluorocannabidiol associated with capsazepine restrains L-DOPA-induced dyskinesia in hemiparkinsonian mice: contribution of anti-inflammatory and anti-glutamatergic mechanisms. Neuropharmacology 251:109926. doi: 10.1016/j.neuropharm.2024.109926, 38554815 PMC11988267

[ref33] Feng L. Lo H. You H. Wu W. Cheng X. Xin J. . (2023). Loss of cannabinoid receptor 2 promotes α-Synuclein-induced microglial synaptic pruning in nucleus accumbens by modulating the pCREB-c-Fos signaling pathway and complement system. Exp. Neurol. 359:114230. doi: 10.1016/j.expneurol.2022.114230, 36162511

[ref34] Finn D. P. Haroutounian S. Hohmann A. G. Krane E. Soliman N. Rice A. S. C. (2021). Cannabinoids, the endocannabinoid system, and pain: a review of preclinical studies. Pain 162, S5–S25. doi: 10.1097/j.pain.0000000000002268, 33729211 PMC8819673

[ref35] Flores-Soto M. E. Corona-Angeles J. A. Tejeda-Martinez A. R. Flores-Guzman P. A. Luna-Mujica I. Chaparro-Huerta V. . (2021). β-Caryophyllene exerts protective antioxidant effects through the activation of NQO1 in the MPTP model of Parkinson’s disease. Neurosci. Lett. 742:135534. doi: 10.1016/j.neulet.2020.135534, 33271195

[ref36] Galán-Ganga M. Del Río R. Jiménez-Moreno N. Díaz-Guerra M. Lastres-Becker I. (2020). Cannabinoid CB2 receptor modulation by the transcription factor NRF2 is specific in microglial cells. Cell. Mol. Neurobiol. 40, 167–177. doi: 10.1007/s10571-019-00719-y, 31385133 PMC11449021

[ref37] Galán-Ganga M. Rodríguez-Cueto C. Merchán-Rubira J. Hernández F. Ávila J. Posada-Ayala M. . (2021). Cannabinoid receptor CB2 ablation protects against TAU induced neurodegeneration. Acta Neuropathol. Commun. 9:90. doi: 10.1186/s40478-021-01196-5, 34001284 PMC8130522

[ref38] Gallego-Landin I. García-Baos A. Castro-Zavala A. Valverde O. (2021). Reviewing the role of the endocannabinoid system in the pathophysiology of depression. Front. Pharmacol. 12:762738. doi: 10.3389/fphar.2021.762738, 34938182 PMC8685322

[ref39] Gao C. Jiang J. Tan Y. Chen S. (2023). Microglia in neurodegenerative diseases: mechanism and potential therapeutic targets. Sig Transduct Target Ther 8:359. doi: 10.1038/s41392-023-01588-0, 37735487 PMC10514343

[ref40] Gasparyan A. Navarrete F. Navarro D. Manzanares J. (2023). Cannabidiol regulates behavioral and brain alterations induced by spontaneous alcohol withdrawal. Neuropharmacology 233:109549. doi: 10.1016/j.neuropharm.2023.109549, 37085012

[ref41] Gioé-Gallo C. Ortigueira S. Prieto-Díaz R. Contino M. Azuaje J. Perrone M. G. . (2025). Conformational restriction of designer drugs reveals subtype-selective and biased CB(2) agonists with neuroprotective effects. J. Med. Chem. 68, 17103–17129. doi: 10.1021/acs.jmedchem.5c00604, 40796509 PMC12406204

[ref42] Gonzalo-Consuegra C. Santos-García I. García-Toscano L. Martín-Baquero R. Rodríguez-Cueto C. Wittwer M. B. . (2024). Involvement of CB(1) and CB(2) receptors in neuroprotective effects of cannabinoids in experimental TDP-43 related frontotemporal dementia using male mice. Biomed. Pharmacother. 174:116473. doi: 10.1016/j.biopha.2024.116473 38522237

[ref43] Grabon W. Bodennec J. Rheims S. Belmeguenai A. Bezin L. (2023a). Update on the controversial identity of cells expressing cnr2 gene in the nervous system. CNS Neurosci. Ther. 29, 760–770. doi: 10.1111/cns.13977, 36604187 PMC9928557

[ref44] Grabon W. Rheims S. Smith J. Bodennec J. Belmeguenai A. Bezin L. (2023b). CB2 receptor in the CNS: from immune and neuronal modulation to behavior. Neurosci. Biobehav. Rev. 150:105226. doi: 10.1016/j.neubiorev.2023.105226, 37164044

[ref45] Grabon W. Ruiz A. Gasmi N. Degletagne C. Georges B. Belmeguenai A. . (2024). CB2 expression in mouse brain: from mapping to regulation in microglia under inflammatory conditions. J. Neuroinflammation 21:206. doi: 10.1186/s12974-024-03202-8, 39160534 PMC11334370

[ref46] He X.-F. Yang X.-F. Li G. Zhao Y. Luo J. Xu J.-H. . (2025). Physical exercise improves the neuronal function in ischemic stroke via microglial CB(2)R/P2Y12 signaling. Mol. Neurobiol. 62, 2039–2057. doi: 10.1007/s12035-024-04391-2, 39066973

[ref47] Hen-Shoval D. Moshe L. Indig-Naimer T. Mechoulam R. Shoval G. Zalsman G. . (2023). Cannabinoid receptor 2 blockade prevents anti-depressive-like effect of Cannabidiol acid methyl Ester in female WKY rats. Int. J. Mol. Sci. 24:3828. doi: 10.3390/ijms24043828, 36835237 PMC9958868

[ref48] Hipp M. S. Kasturi P. Hartl F. U. (2019). The proteostasis network and its decline in ageing. Nat. Rev. Mol. Cell Biol. 20, 421–435. doi: 10.1038/s41580-019-0101-y, 30733602

[ref49] Hu F. Lei Y. Han R. Yang B. Liang Y. Luan J. . (2025). The efficacy and pharmacological mechanism of Guilingji to prevent Alzheimer’s disease. Alzheimer's Res. Ther. 17:157. doi: 10.1186/s13195-025-01790-y, 40660408 PMC12257861

[ref50] Jantsch J. Rodrigues F. d. S. Wickert F. Fraga G. d. F. Dias V. S. Bitencourt Y. M. . (2025). Cannabidiol attenuates diet-induced metabolic endotoxemia, neuroinflammation, and anxiety-like behaviors in male aged rats. Brain Behav. Immun. 130:106121. doi: 10.1016/j.bbi.2025.106121 41022293

[ref51] Joers V. Murray B. C. McLaughlin C. Oliver D. Staley H. E. Coronado J. . (2024). Modulation of cannabinoid receptor 2 alters neuroinflammation and reduces formation of alpha-synuclein aggregates in a rat model of nigral synucleinopathy. J. Neuroinflammation 21:240. doi: 10.1186/s12974-024-03221-5, 39334169 PMC11438102

[ref52] Johnson A. L. Verbitsky R. Hudson J. Dean R. Hamilton T. J. (2023). Cannabinoid type-2 receptors modulate terpene induced anxiety-reduction in zebrafish. Biomed. Pharmacother. 168:115760. doi: 10.1016/j.biopha.2023.115760 37865998

[ref53] Karan A. A. Spivak Y. S. Suleymanova E. M. Gerasimov K. A. Bolshakov A. P. Vinogradova L. V. (2024). Distant neuroinflammation acutely induced by focal brain injury and its control by endocannabinoid system. Exp. Neurol. 373:114679. doi: 10.1016/j.expneurol.2024.114679, 38190933

[ref54] Kibret B. G. Roberts A. Kneebone A. Embaby S. Fernandez J. Liu Q.-R. . (2023). Cannabinoid CB2 receptors modulate alcohol induced behavior, and neuro-immune dysregulation in mice. Behav. Brain Res. 448:114439. doi: 10.1016/j.bbr.2023.114439, 37061199

[ref55] Komorowska-Müller J. A. Schmöle A.-C. (2020). CB2 receptor in microglia: the guardian of self-control. Int. J. Mol. Sci. 22:19. doi: 10.3390/ijms22010019, 33375006 PMC7792761

[ref56] Kordinová H. Mik V. Hemelíková N. Gonzalez G. Štěpánková Š. Górová V. . (2026). Development of 2,6,9-trisubstituted purines as neuroprotective agents targeting butyrylcholinesterase and cannabinoid CB2 receptor. Eur. J. Med. Chem. 301:118217. doi: 10.1016/j.ejmech.2025.118217, 41037984

[ref57] Kruk-Slomka M. Dzik A. Biala G. (2025). The effects of indirect and direct modulation of endocannabinoid system function on anxiety-related behavior in mice assessed in the elevated plus maze test. Molecules 30:867. doi: 10.3390/molecules30040867, 40005177 PMC11857936

[ref58] Laloli K. J. Rentsch P. Stayte S. Vissel B. (2025). Novel floxed cannabinoid receptor 2 mouse line combines knockout capability with dual fluorescent reporters. Front. Pharmacol. 16:1682979. doi: 10.3389/fphar.2025.1682979, 41347158 PMC12672438

[ref59] Li S. Yang P. Wu Z. Huang W. Zhu X. Zhong L. (2024). The effects and mechanisms of AM1241 in alleviating cerebral ischemia-reperfusion injury. Brain Res. Bull. 215:111025. doi: 10.1016/j.brainresbull.2024.111025, 38964663

[ref60] Li M. Ye X. Huang Z. Ye L. Chen C. (2025). Global burden of Parkinson’s disease from 1990 to 2021: a population-based study. BMJ Open 15:e095610. doi: 10.1136/bmjopen-2024-095610, 40288800 PMC12035419

[ref61] Liu M. Pan D. Wang M. Deng H. Ma Z. (2024). JWH133 attenuates behavior deficits and iron accumulation in 6-OHDA-induced Parkinson’s disease model rats. J. Neurophysiol. 132, 733–743. doi: 10.1152/jn.00137.2024, 39015077

[ref62] Lu H.-C. Mackie K. (2021). Review of the endocannabinoid system. Biol. Psychiatry: Cognit. Neurosci. Neuroimaging 6, 607–615. doi: 10.1016/j.bpsc.2020.07.016, 32980261 PMC7855189

[ref63] Lu S. Zhang S. Oung S. Diedrich J. K. Han P. Arnold-Garcia O. . (2025). TDP-43 skein-like inclusions are formed by BAG3- and HSP70-guided co-aggregation with actin-binding proteins. Nat. Cell Biol. 27, 1925–1937. doi: 10.1038/s41556-025-01789-5, 41174004 PMC12782899

[ref64] Lui F. Tsao J. W. (2026). “Alzheimer Disease,” in StatPearls, (Treasure Island (FL): StatPearls Publishing). Available online at: http://www.ncbi.nlm.nih.gov/books/NBK499922/ (Accessed April 15, 2026).

[ref65] Malkesman O. Braw Y. Maayan R. Weizman A. Overstreet D. H. Shabat-Simon M. . (2006). Two different putative genetic animal models of childhood depression. Biol. Psychiatry 59, 17–23. doi: 10.1016/j.biopsych.2005.05.039, 16095569

[ref66] Mallmann M. P. Mello F. K. Neuberger B. da Costa Sobral K. G. Fighera M. R. Royes L. F. F. . (2022). Beta-caryophyllene attenuates short-term recurrent seizure activity and blood-brain-barrier breakdown after pilocarpine-induced status epilepticus in rats. Brain Res. 1784:147883. doi: 10.1016/j.brainres.2022.147883, 35300975

[ref67] Matias M. E. Radulski D. R. Rodrigues da Silva T. Raymundi A. M. Stern C. A. J. Zampronio A. R. (2023). Involvement of cannabinoid receptors and neuroinflammation in early sepsis: implications for posttraumatic stress disorder. Int. Immunopharmacol. 123:110745. doi: 10.1016/j.intimp.2023.110745, 37541107

[ref68] Meanti R. Bresciani E. Rizzi L. Molteni L. Coco S. Omeljaniuk R. J. . (2025). Cannabinoid receptor 2 (CB2R) as potential target for the pharmacological treatment of neurodegenerative diseases. Biomed. Pharmacother. 186:118044. doi: 10.1016/j.biopha.2025.118044, 40209306

[ref69] Medina-Vera D. Zhao H. Bereczki E. Rosell-Valle C. Shimozawa M. Chen G. . (2023). The expression of the endocannabinoid receptors CB2 and GPR55 is highly increased during the progression of Alzheimer’s disease in app(NL-G-F) knock-in mice. Biology 12:805. doi: 10.3390/biology12060805, 37372090 PMC10295339

[ref70] Moe A. Rayasam A. Sauber G. Shah R. K. Doherty A. Yuan C.-Y. . (2024). Type 2 cannabinoid receptor expression on microglial cells regulates neuroinflammation during graft-versus-host disease. J. Clin. Invest. 134:e175205. doi: 10.1172/JCI175205, 38662453 PMC11142740

[ref71] Montalesi E. Caissutti D. Moliterni C. Ferrante A. Pepponi R. Garofalo T. . (2025). Proteostasis network response to environmental chronic stress: linking survival to protein aggregation in a human neuroblastoma cellular model. Cell. Mol. Life Sci. 82:430. doi: 10.1007/s00018-025-05884-6, 41307665 PMC12660585

[ref72] Müller L. Di Benedetto S. Müller V. (2025). The dual nature of neuroinflammation in networked brain. Front. Immunol. 16:1659947. doi: 10.3389/fimmu.2025.1659947, 40909282 PMC12404926

[ref73] Navarrete F. Cabrera-Rubio R. Gasparyan A. Aarnio R. López-Picón F. Helin S. . (2025). Cannabidiol modulates brain molecular alterations, gut microbiota dysbiosis and alcohol self-administration in a mouse model of fetal alcohol spectrum disorder. Biomed. Pharmacother. 193:118791. doi: 10.1016/j.biopha.2025.118791, 41273930

[ref74] Otte C. Gold S. M. Penninx B. W. Pariante C. M. Etkin A. Fava M. . (2016). Major depressive disorder. Nat. Rev. Dis. Primers 2:16065. doi: 10.1038/nrdp.2016.65, 27629598

[ref75] Pacheco-Sánchez B. Tovar R. Ben Rabaa M. Sánchez-Salido L. Vargas A. Suárez J. . (2023). Sex-dependent altered expression of cannabinoid signaling in hippocampal astrocytes of the triple transgenic mouse model of Alzheimer’s disease: implications for controlling Astroglial activity. Int. J. Mol. Sci. 24:12598. doi: 10.3390/ijms241612598, 37628778 PMC10454447

[ref76] Paredes-Ruiz K. J. Chavira-Ramos K. Galvan-Arzate S. Rangel-López E. Karasu Ç. Túnez I. . (2023). Monoacylglycerol lipase inhibition prevents short-term mitochondrial dysfunction and oxidative damage in rat brain Synaptosomal/mitochondrial fractions and cortical slices: role of cannabinoid receptors. Neurotox. Res. 41, 514–525. doi: 10.1007/s12640-023-00661-4, 37458923

[ref77] Pertwee R. G. (2012). Targeting the endocannabinoid system with cannabinoid receptor agonists: pharmacological strategies and therapeutic possibilities. Philos. Trans. R. Soc. B 367, 3353–3363. doi: 10.1098/rstb.2011.0381, 23108552 PMC3481523

[ref78] Picone R. P. Kendall D. A. (2015). Minireview: from the bench, toward the clinic: therapeutic opportunities for cannabinoid receptor modulation. Mol. Endocrinol. 29, 801–813. doi: 10.1210/me.2015-1062, 25866875 PMC4447638

[ref79] Poewe W. Seppi K. Tanner C. M. Halliday G. M. Brundin P. Volkmann J. . (2017). Parkinson disease. Nat. Rev. Dis. Primers 3:17013. doi: 10.1038/nrdp.2017.13, 28332488

[ref80] Ramirez S. H. Haskó J. Skuba A. Fan S. Dykstra H. McCormick R. . (2012). Activation of cannabinoid receptor 2 attenuates leukocyte–endothelial cell interactions and blood–brain barrier dysfunction under inflammatory conditions. J. Neurosci. 32, 4004–4016. doi: 10.1523/JNEUROSCI.4628-11.2012, 22442067 PMC3325902

[ref81] Rathod S. S. Agrawal Y. O. (2025). Β-Caryophyllene (CB2 agonist) mitigates rotenone-induced neurotoxicity and apoptosis in SH-SY5Y neuroblastoma cells via modulation of GSK-3β/NRF2/HO-1 axis. Naunyn Schmiedeberg's Arch. Pharmacol. 398, 15943–15963. doi: 10.1007/s00210-025-04281-x, 40410551

[ref82] Reyes-Resina I. Lillo J. Raïch I. Rebassa J. B. Capó T. Badia P. . (2025). The interplay between CB(2) and NMDA receptors in Parkinson’s disease. Int. J. Mol. Sci. 26:9419. doi: 10.3390/ijms26199419, 41096688 PMC12524789

[ref83] Ricardi C. Mazzierli A. Guglielmo S. Origlia N. Gado F. Manera C. . (2025). Multi-target protective effects of β-Caryophyllene (BCP) at the intersection of Neuroinflammation and neurodegeneration. Int. J. Mol. Sci. 26:6027. doi: 10.3390/ijms26136027, 40649806 PMC12249661

[ref84] Riku Y. Yoshida M. Iwasaki Y. Sobue G. Katsuno M. Ishigaki S. (2022). TDP-43 Proteinopathy and Tauopathy: do they have Pathomechanistic links? IJMS 23:15755. doi: 10.3390/ijms232415755, 36555399 PMC9779029

[ref85] Roberts A. Christian M. Dilone L. N. Nelson N. Endrino M. J. Kneebone A. . (2023). Alcohol induced behavioral and immune perturbations are attenuated by activation of CB2 cannabinoid receptors. Adv. Drug Alcohol Res. 3:11602. doi: 10.3389/adar.2023.11602, 38389814 PMC10880753

[ref86] Rodrigues F. S. Newton W. R. Tassinari I. D. da Cunha Xavier F. H. Marx A. de Fraga L. S. . (2024). Cannabidiol prevents LPS-induced inflammation by inhibiting the NLRP3 inflammasome and iNOS activity in BV2 microglia cells via CB2 receptors and PPARγ. Neurochem. Int. 177:105769. doi: 10.1016/j.neuint.2024.105769, 38761855

[ref87] Rosenzweig S. Carmichael S. T. (2015). The axon-glia unit in white matter stroke: mechanisms of damage and recovery. Brain Res. 1623, 123–134. doi: 10.1016/j.brainres.2015.02.019, 25704204 PMC4545468

[ref88] Sadleir K. R. Kandalepas P. C. Buggia-Prévot V. Nicholson D. A. Thinakaran G. Vassar R. (2016). Presynaptic dystrophic neurites surrounding amyloid plaques are sites of microtubule disruption, BACE1 elevation, and increased aβ generation in Alzheimer’s disease. Acta Neuropathol. 132, 235–256. doi: 10.1007/s00401-016-1558-9, 26993139 PMC4947125

[ref89] Scarlett K. A. White E.-S. Z. Coke C. J. Carter J. R. Bryant L. K. Hinton C. V. (2018). Agonist-induced CXCR4 and CB2 Heterodimerization inhibits Gα13/RhoA-mediated migration. Mol. Cancer Res. 16, 728–739. doi: 10.1158/1541-7786.MCR-16-0481, 29330286 PMC5882517

[ref90] Schwab E. D. P. Queiroz R. Fiebrantz A. K. B. Bastos M. Bonini J. S. Silva W. C. F. N. (2022). Hypothesis on ontogenesis and pathophysiology of Alzheimer’s disease. Einstein 20:eRW0170. doi: 10.31744/einstein_journal/2022RW0170, 36449761 PMC9744435

[ref91] Seedat S. Scott K. M. Angermeyer M. C. Berglund P. Bromet E. J. Brugha T. S. . (2009). Cross-National Associations between Gender and mental disorders in the World Health Organization world mental health surveys. Arch. Gen. Psychiatry 66:785. doi: 10.1001/archgenpsychiatry.2009.36, 19581570 PMC2810067

[ref92] Shan R. Zhang Y. Shi Y. Wang X. Wang X. Ma G. . (2024). Activation of cannabinoid type 2 receptor in microglia reduces Neuroinflammation through inhibiting aerobic glycolysis to relieve hypertension. Biomolecules 14:333. doi: 10.3390/biom14030333, 38540753 PMC10967819

[ref93] Silva-Llanes I. Rodríguez-López S. González-Naranjo P. Sastre E. D. López M. G. Páez J. A. . (2025). Targeting CB2 receptor with a novel antagonist reverses cognitive decline, neurodegeneration and pyroptosis in a TAU-dependent frontotemporal dementia mouse model. Brain Behav. Immun. 127, 251–268. doi: 10.1016/j.bbi.2025.03.008 40081780

[ref94] Simard M. Rakotoarivelo V. Di Marzo V. Flamand N. (2022). Expression and functions of the CB2 receptor in human leukocytes. Front. Pharmacol. 13:826400. doi: 10.3389/fphar.2022.826400, 35273503 PMC8902156

[ref95] Sobue A. Komine O. Endo F. Kakimi C. Miyoshi Y. Kawade N. . (2024). Microglial cannabinoid receptor type II stimulation improves cognitive impairment and neuroinflammation in Alzheimer’s disease mice by controlling astrocyte activation. Cell Death Dis. 15:858. doi: 10.1038/s41419-024-07249-6, 39587077 PMC11589152

[ref96] Soethoudt M. Grether U. Fingerle J. Grim T. W. Fezza F. De Petrocellis L. . (2017). Cannabinoid CB2 receptor ligand profiling reveals biased signalling and off-target activity. Nat. Commun. 8:13958. doi: 10.1038/ncomms13958, 28045021 PMC5216056

[ref97] Soliman N. Haroutounian S. Hohmann A. G. Krane E. Liao J. Macleod M. . (2021). Systematic review and meta-analysis of cannabinoids, cannabis-based medicines, and endocannabinoid system modulators tested for antinociceptive effects in animal models of injury-related or pathological persistent pain. Pain 162, S26–S44. doi: 10.1097/j.pain.0000000000002269, 33729209 PMC8216112

[ref98] Spatz P. Steinmüller S. A. M. Tutov A. Poeta E. Morilleau A. Carles A. . (2023). Dual-acting small molecules: subtype-selective cannabinoid receptor 2 agonist/Butyrylcholinesterase inhibitor hybrids show neuroprotection in an Alzheimer’s disease mouse model. J. Med. Chem. 66, 6414–6435. doi: 10.1021/acs.jmedchem.3c00541, 37127287 PMC10184129

[ref99] Stampanoni Bassi M. Gilio L. Maffei P. Dolcetti E. Bruno A. Buttari F. . (2018). Exploiting the multifaceted effects of cannabinoids on mood to boost their therapeutic use against anxiety and depression. Front. Mol. Neurosci. 11:424. doi: 10.3389/fnmol.2018.00424, 30515077 PMC6256035

[ref100] Standoli S. Rapino C. Di Meo C. Rudowski A. Kämpfer-Kolb N. Volk L. M. . (2023). Sphingosine kinases at the intersection of pro-inflammatory LPS and anti-inflammatory endocannabinoid signaling in BV2 mouse microglia cells. Int. J. Mol. Sci. 24:8508. doi: 10.3390/ijms24108508, 37239854 PMC10217805

[ref101] Starr A. Rathore S. Daniali M. Gaskill P. J. Akay-Espinoza C. Jordan-Sciutto K. L. (2025). Differential effects of cannabinoid receptor 2 agonists on HIV replication and inflammatory activation in monocyte-derived macrophages and induced pluripotent stem cell-derived microglia. J. Neuroimmune Pharmacol. 20:87. doi: 10.1007/s11481-025-10254-x, 41075102 PMC12515222

[ref102] Tan R. H. Yang Y. Kim W. S. Dobson-Stone C. Kwok J. B. Kiernan M. C. . (2017). Distinct TDP-43 inclusion morphologies in frontotemporal lobar degeneration with and without amyotrophic lateral sclerosis. Acta Neuropathol. Commun. 5:76. doi: 10.1186/s40478-017-0480-2, 29078806 PMC5658959

[ref103] Tanaka K. Matsuda N. (2014). Proteostasis and neurodegeneration: the roles of proteasomal degradation and autophagy. Biochim. Biophys. Acta 1843, 197–204. doi: 10.1016/j.bbamcr.2013.03.012, 23523933

[ref104] Targa A. D. S. Dos Santos-Lima G. Z. Rodrigues L. S. Cavalcante S. F. Fontenele-Araújo J. Torterolo P. . (2025). The cannabinoid CB2 receptor: improvement of sleep or memory in rotenone model of Parkinson's disease. Eur. J. Pharmacol. 1000:177745. doi: 10.1016/j.ejphar.2025.177745 40383223

[ref105] Tisi A. Scipioni L. Carozza G. Di Re L. Cimino G. Di Meo C. . (2025). Alterations of endocannabinoid signaling and microglia reactivity in the retinas of AD-like mice precede the onset of hippocampal β-amyloid plaques. J. Neurochem. 169:e16256. doi: 10.1111/jnc.16256, 39556462 PMC11808635

[ref106] Toledano R. S. Akirav I. (2025). Cannabidiol prevents cognitive and social deficits in a male rat model of Alzheimer’s disease through CB1 activation and inflammation modulation. Neuropsychopharmacology 50, 1916–1927. doi: 10.1038/s41386-025-02213-0, 40859005 PMC12603115

[ref107] Uzuneser T. C. Szkudlarek H. J. Jones M. J. Nashed M. G. Clement T. Wang H. . (2023). Identification of a novel fatty acid binding protein-5-CB2 receptor-dependent mechanism regulating anxiety behaviors in the prefrontal cortex. Cerebral cortex (New York, N.Y: Oxford University Press). 33, 2470–2484. doi:10.1093/cercor/bhac220, 35650684 PMC10016066

[ref108] Van Sickle M. D. Duncan M. Kingsley P. J. Mouihate A. Urbani P. Mackie K. . (2005). Identification and functional characterization of brainstem cannabinoid CB2 receptors. Science 310, 329–332. doi: 10.1126/science.1115740 16224028

[ref109] Videtta G. Sasia C. Quadrino S. Brighenti V. Bertarini L. Caroli C. . (2026). A sesquiterpene-rich essential oil from *Cannabis sativa* L. attenuates symptoms and neuroinflammation in experimental autoimmune encephalomyelitis model through a CB2-mediated signalling. Phytomed. Int. J. Phytother. Phytopharm. 155:158068. doi: 10.1016/j.phymed.2026.158068, 41875735

[ref110] Wang Y. Cao M. Zhang Y. Chen Q. Chen Z. Jia J. (2024). The CB2-PKC pathway is involved in esketamine-induced anti-inflammation in BV-2 microglial cells exposed to lipopolysaccharides. Am. J. Transl. Res. 16, 4466–4478. doi: 10.62347/RRZF5229, 39398580 PMC11470345

[ref111] Wang J. Du H. Li M. Yan T. Jia Y. (2025). Schisandra chinensis lignans exerts endocannabinoids-like antidepressive effect: the phagocytotic relationship of activated CB2R-mediated M2 microglia and “stressed-but-viable” neuron. J. Ethnopharmacol. 342:119385. doi: 10.1016/j.jep.2025.119385, 39832627

[ref112] Wang Y. Liu G. Hong D. Chen F. Ji X. Cao G. (2016). White matter injury in ischemic stroke. Prog. Neurobiol. 141, 45–60. doi: 10.1016/j.pneurobio.2016.04.005, 27090751 PMC5677601

[ref113] Wang M. Liu M. Ma Z. (2023). Cannabinoid type 2 receptor activation inhibits MPP(+)-induced M1 differentiation of microglia through activating PI3K/Akt/Nrf2 signal pathway. Mol. Biol. Rep. 50, 4423–4433. doi: 10.1007/s11033-023-08395-4, 36977807

[ref114] Whiting P. F. Wolff R. F. Deshpande S. Di Nisio M. Duffy S. Hernandez A. V. . (2015). Cannabinoids for medical use: a systematic review and meta-analysis. JAMA 313:2456. doi: 10.1001/jama.2015.6358 26103030

[ref115] Wu X. Wu Y. Tang F. Wang Y. Li C. Wu S. . (2025). Foxq1 activates CB2R with oleamide to alleviate POCD. Brain Pathol. 35:e13289. doi: 10.1111/bpa.13289, 39046224 PMC11669408

[ref116] Xia X. Li K. Zou W. Wang L. (2025). The central role of microglia in major depressive disorder and its potential as a therapeutic target. Front. Behav. Neurosci. 19:1598178. doi: 10.3389/fnbeh.2025.1598178, 40881472 PMC12381880

[ref117] Xin Q. Xu F. Ma Z. Wu J. (2024). β-Caryophyllene mitigates ischemic stroke-induced white matter lesions by inhibiting pyroptosis. Exp. Cell Res. 442:114214. doi: 10.1016/j.yexcr.2024.114214 39159913

[ref118] Yang W. Gong X. Sun H. Wu C. Suo J. Ji J. . (2024). Discovery of a CB(2) and 5-HT(1A) receptor dual agonist for the treatment of depression and anxiety. Eur. J. Med. Chem. 265:116048. doi: 10.1016/j.ejmech.2023.116048 38150961

[ref119] Yao B. B. Mukherjee S. Fan Y. Garrison T. R. Daza A. V. Grayson G. K. . (2006). In vitro pharmacological characterization of AM1241: a protean agonist at the cannabinoid CB2 receptor? Br. J. Pharmacol. 149, 145–154. doi: 10.1038/sj.bjp.0706838, 16894349 PMC2013801

[ref120] Yu T. Zhang X. Shi H. Tian J. Sun L. Hu X. . (2019). P2Y12 regulates microglia activation and excitatory synaptic transmission in spinal lamina II neurons during neuropathic pain in rodents. Cell Death Dis. 10:165. doi: 10.1038/s41419-019-1425-4, 30778044 PMC6379416

[ref121] Zhang Y. Huang Q. Wang S. Liao Z. Jin H. Huang S. . (2022). The food additive β-Caryophyllene exerts its neuroprotective effects through the JAK2-STAT3-BACE1 pathway. Front. Aging Neurosci. 14:814432. doi: 10.3389/fnagi.2022.814432, 35296033 PMC8919047

[ref122] Zhang H.-Y. Shen H. Gao M. Ma Z. Hempel B. J. Bi G.-H. . (2021). Cannabinoid CB2 receptors are expressed in glutamate neurons in the red nucleus and functionally modulate motor behavior in mice. Neuropharmacology 189:108538. doi: 10.1016/j.neuropharm.2021.108538, 33789118 PMC8122071

[ref123] Zhao H. Deng L. Chen S. Wang X. Dong Z. (2023). Neuroprotection of β-caryophyllene against cerebral ischemia/reperfusion injury by inhibiting P38 MAPK/NLRP3 signaling pathway. Neuroreport 34, 617–623. doi: 10.1097/WNR.0000000000001932, 37395187

[ref124] Zhao S. Liu S. Gong Y. Ma Z. (2025). The effect of cannabinoid type II receptor on the excitability of substantia nigra dopaminergic neurons. Front. Pharmacol. 16:1522210. doi: 10.3389/fphar.2025.1522210, 40028168 PMC11867961

[ref125] Zhu Y. Huang R. Wang D. Yu L. Liu Y. Huang R. . (2023). EVs-mediated delivery of CB2 receptor agonist for Alzheimer’s disease therapy. Asian J. Pharm. Sci. 18:100835. doi: 10.1016/j.ajps.2023.100835, 37645682 PMC10460952

[ref126] Zhu H. Xiao F. Xiao Y. Guo Y. Shan X. Zhang Z. . (2023). Targeting CB2R in astrocytes for Parkinson’s disease therapy: unraveling the Foxg1-mediated neuroprotective mechanism through autophagy-mediated NLRP3 degradation. J. Neuroinflammation 20:304. doi: 10.1186/s12974-023-02989-2, 38110963 PMC10729372

